# Two-dimensional shape memory graphene oxide

**DOI:** 10.1038/ncomms11972

**Published:** 2016-06-21

**Authors:** Zhenyue Chang, Junkai Deng, Ganaka G. Chandrakumara, Wenyi Yan, Jefferson Zhe Liu

**Affiliations:** 1 Department of Mechanical and Aerospace Engineering, Monash University, Clayton, Victoria 3800, Australia; 2 Monash Centre for Atomically Thin Materials, Monash University, Clayton, Victoria 3800, Australia; 3 State Key Laboratory for Mechanical Behaviour of Materials, Xi'an Jiaotong University, Xi'an 710049, China

## Abstract

Driven by the increasing demand for micro-/nano-technologies, stimuli-responsive shape memory materials at nanoscale have recently attracted great research interests. However, by reducing the size of conventional shape memory materials down to approximately nanometre range, the shape memory effect diminishes. Here, using density functional theory calculations, we report the discovery of a shape memory effect in a two-dimensional atomically thin graphene oxide crystal with ordered epoxy groups, namely C_8_O. A maximum recoverable strain of 14.5% is achieved as a result of reversible phase transition between two intrinsically stable phases. Our calculations conclude co-existence of the two stable phases in a coherent crystal lattice, giving rise to the possibility of constructing multiple temporary shapes in a single material, thus, enabling highly desirable programmability. With an atomic thickness, excellent shape memory mechanical properties and electric field stimulus, the discovery of a two-dimensional shape memory graphene oxide opens a path for the development of exceptional micro-/nano-electromechanical devices.

## Abstract (web summary)

When reducing the size of shape memory materials to the nanoscale regime, the memory effect tends to diminish. Here, the authors report a theoretical proposal of a shape memory graphene oxide with ordered epoxy groups retaining excellent programmability and actuation capabilities.

## Graphical abstract

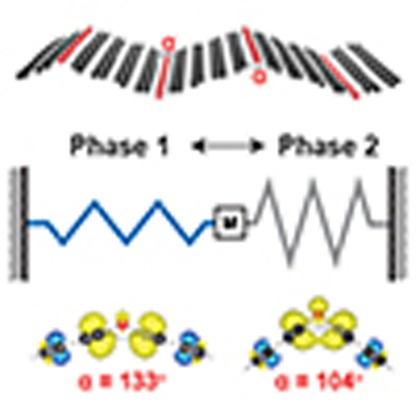

Shape memory materials (SMMs) are featured by the ability to recover their original shape from a significant quasi-plastic deformation upon an appropriate external stimulus^1^. This interesting shape memory phenomenon occurs as a result of reversible phase transition, known as the shape memory effect (SME)^2^. The recoverable mechanical deformation from SMMs, for example, ∼0.5–8% strain from shape memory alloys (SMAs)^3^, 10% strain from shape memory ceramics (SMCs)^4^ and up to 400% strain from shape memory polymers (SMPs)^5^, endows them with an indispensable role in various applications such as actuators, sensors, mechanical joints, dampers and morphing devices^3^,^5^,^6^,^7^,^8^. More interestingly, the most important feature of SMM, namely programmability, has been explored as a novel device design concept, so-called material-as-machine^9^, in which SMM can be programmed for actuation/motion following a pre-determined sequence, just like machines but with greater intelligence and flexibility whereby the material can sense and react accordingly^10^. This is particularly desirable for mechanical devices at a smaller size scale.

Indeed, recent interest and utilization of conventional SMMs (that is, SMAs, SMPs and SMCs) are motivated by the increasing popularity and large potential associated with micro- and nanotechnologies. Several studies have successfully demonstrated micro-/nanoscopic SME by introducing micro-/nanostructures at the surfaces of bulk SMAs and SMPs^11^,^12^,^13^. Nevertheless, it should be noted that the materials themselves are still at relatively large scales (for example, macroscopic or sub-micron). Further miniaturization of conventional SMMs down to the nanometre range is hampered by their inherent constraints. For instance, recent studies on nanometre-sized SMA demonstrated that martensitic phase transformation (physical origin of the SME) completely diminishes below a critical size (4–60 nm), resulting from the influence of surface and interface energy^14^,^15^,^16^,^17^. SMAs also experience other issues, such as oxidation and instability at nanoscale. In SMC film, the free charges at the surface lead to a depolarized electric field (**E**-field). It has been reported that by reducing the film thickness below a critical value (for example, ∼20 nm), SMCs lose ferroelectricity, thus, the SME^18^. To date, there has been no reported study regarding SMPs down to the nanometre range, which could be attributed to fabrication challenges at this scale. In addition, the requirement of thermal stimulation that is commonly employed with conventional SMMs could restrict their implementation in nano-devices because of difficulties of precise control of the heating and cooling process and a low actuation frequency (about 1 Hz)^3^.

Here, using first-principles density functional theory (DFT) calculations, we report the discovery of SME in a two-dimensional atomically thin graphene oxide (GO) crystal (namely C_8_O), triggered by a combination of **E**-field and mechanical force stimuli. In depth analysis reveals a unique intra-molecular chemical interaction between oxygen *sp*^2^ lone pair (*lp*) and carbon *π* orbitals as the structural origin for the SME. We also theoretically demonstrate the programmability (a key attribute of SMM) of C_8_O, enabling C_8_O as an advanced SMM at nanometre scale. This shape memory C_8_O has superior shape memory mechanical properties well above the upper bound drawn in a recent survey of mechanical properties among available SMMs^19^. It is noteworthy that the **E**-field and mechanical force stimuli could overcome the limitations of commonly employed thermal stimuli at small size scales.

## Results

### Bi-stable phases of C_8_O with ordered epoxy groups

GO inherits a diverse range of crystal structures with excellent physical, chemical and mechanical properties. Such outstanding properties of GO have stimulated researchers to investigate its usage in a broad range of applications, such as energy storage, filtration membranes, actuators, sensors and transistors^20^,^21^,^22^,^23^,^24^,^25^,^26^,^27^,^28^. Extensive studies in last decade identified epoxy and hydroxyl groups as two major functional groups on the basal plane of GO^29^,^30^. Depends on the choice of synthesis methods under different conditions^31^,^32^,^33^,^34^,^35^,^36^,^37^,^38^,^39^, various GOs structures are observed. For example, hydroxyl groups dominant the GO surface under hydrogen-rich environment^34^. On the other hand, recent experimental progress suggests it is possible to produce GO with only epoxy groups under well-controlled experimental conditions^33^,^36^.

Interestingly, some experimental studies show the existence of ordered line patterns of epoxy groups on the graphene basal plane with various C/O ratio^37^,^38^,^39^. Pandey *et al*. used a scanning tunnelling electron microscope to capture atomic images of highly ordered GO structure with linearly aligned epoxy groups in a region that covers over 50% of the total scanned area^37^. Fujii and Enoki observed regularly spaced line defects on chemically oxidized graphene sheets with length of ∼100 nm using non-contact atomic force microscope, which were identified as the linear arrangement of epoxide groups^38^. Meanwhile, many theoretical studies were carried out to investigate the formation mechanism of the ordered epoxy lines in experiments^39^,^40^,^41^. In general, the presence of epoxy groups severely strains the graphene lattice. A cooperative alignment of epoxy groups in rows can efficiently reduce the strain. In addition, the aligned epoxy groups are able to unzip the C–C bonds beneath the oxygen atoms and reduce the energy further by ∼1.2 eV per bond, which was believed to cause the faulty lines in graphene observed via dark-field optical microscope images^39^.

**Figure 1:**
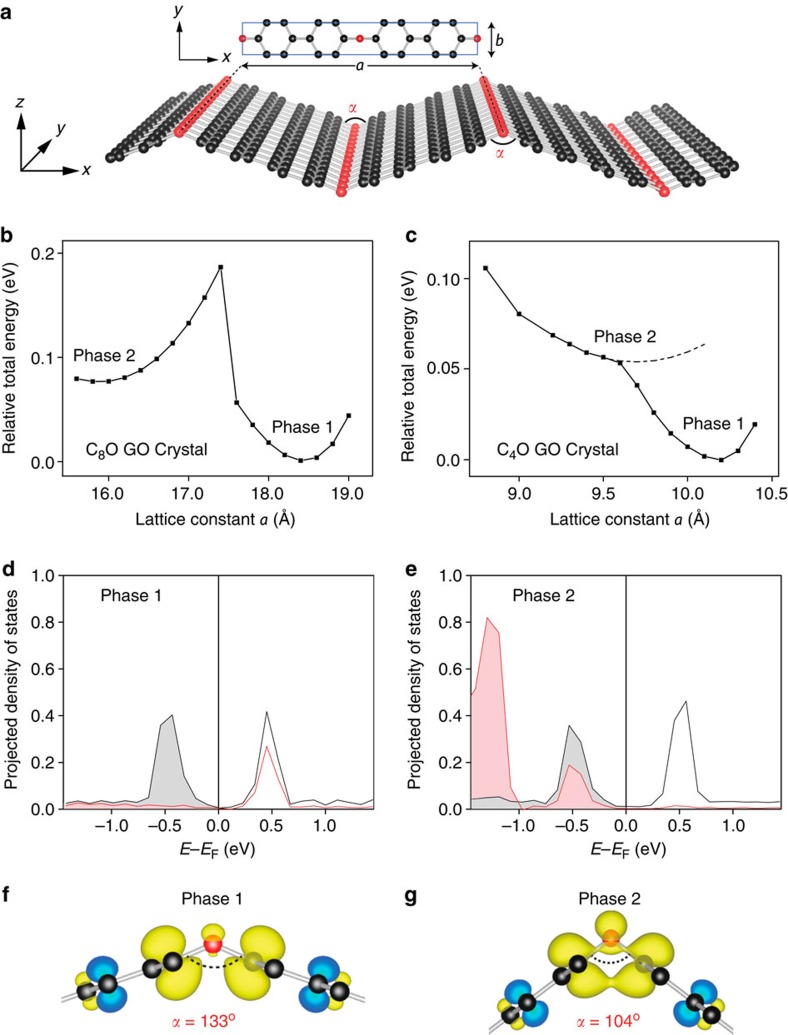
Bi-stable phases of atomically thin C_8_O with ordered epoxy groups. (**a**) Crystal structure of C_8_O with *a* and *b* as the lattice constant in *x* and *y* axes (rectangular supercells are used). Black and red spheres represent the carbon (C) and oxygen (O) atoms, respectively. Angle *α* measures the bond angle of oxygen atom and its two neighbouring carbon atoms. The C_8_O GO crystal unit cell contains 16 carbon atoms and 2 oxygen atoms. (**b**) The relative total energy as a function of lattice constant *a* for C_8_O. The two local minimum points represent the two stable phases: phase 1 and phase 2. The lattice constant *a* of phase 1 and phase 2 C_8_O is 18.384 and 15.718 Å, respectively. The lattice constant in *y*-direction is 2.475 Å for both cases. (**c**) For a comparison, the relative total energy versus lattice constant *a* for a similar C_4_O crystal. (**d**,**e**) The projection density of state (PDOS) results for the two stable phases of C_8_O. The black curves represent p-electrons DOS of the carbon atoms and the red curves represent p-electrons DOS of the oxygen atom. The vertical lines represent the Fermi level (*E*_F_). Below *E*_F_, red- and grey-shaded areas indicate occupation of electrons on the p-orbitals of oxygen and carbon atoms, respectively. (**f**,**g**) Partial charge density surrounding the oxygen and its neighbouring carbon atoms (within the energy range from −1.0 to 0.0 eV with reference to *E*_F_) for both phase 1 and phase 2 C_8_O.

Figure 1a shows an unzipped GO structure that closely resembles the one observed in Fujii's experiments^38^. We studied two different C/O ratio cases, namely C_4_O and C_8_O. The adjacent rows of epoxy groups are placed on opposite sides of graphene basal plane. Such a configuration has been reported to be more stable as compared with the case with epoxy groups on a single side^41^,^42^. In Fig. 1a, lattice constant *a* represents the unit cell length in *x* axis and *α* measures carbon–oxygen–carbon (C–O–C) bond angle of the epoxy group. Intriguingly, the unzipped C_8_O has two local minimum points in the relative total energy as a function of *a*, separated by an energy barrier of ∼100 meV (Fig. 1b). They represent two stable (or bi-stable) phases, denoted as phase 1 (P1) and phase 2 (P2). Besides the lattice constant, the most noticeable structural difference is the C–O–C bond angle *α* equal to 133° for P1 and 104° for P2 in Fig. 1f,g. Both P1 and P2 show the unzipped characteristics, because the C–C bonds underneath the oxygen atoms, having a length greater than 2.2 Å, are clearly ruptured.

Supplementary Fig. 1 shows the phonon density of states (DOSs) for both phases^43^. Based on harmonic approximate, we estimated the free energies of P1 and P2 at 300 K are –158.2925, eV and –158.1958, eV, respectively. P1 is the more stable phase and the free energy difference is 97 meV, moderately increased from 76 meV at zero K (Fig. 1b).

To investigate physical origins of the bi-stable phases, the electronic DOSs of P1 and P2 are shown in Supplementary Fig. 2a,b. There are no obvious differences. A close inspection of the projection density of state (PDOS) of the oxygen atom and its two neighbouring carbon atoms reveals distinctive features near the *E*_F_ in these two phases. Figure 1e and Supplementary Fig. 2 show clearly overlapped density peaks of *s*- and *p*-electrons at –0.5 eV below the *E*_F_ in P2, suggesting chemical interactions among the oxygen and its two neighbouring carbon atoms. In contrast, P1 only shows a *p*-electrons density peak of the carbon atoms near –0.5 eV, instead a similar overlap appears at +0.5 eV above the *E*_F_ (Fig. 1d).

Supplementary Table 1 lists the partial charge density for the oxygen and carbon atoms within an energy range from –20 to 0 eV referenced to *E*_F_. The oxygen atom shows *sp*^2^ hybridization in both phases (Supplementary Note 1). Its two half-filled *sp*^2^ orbitals interact with those of carbon atoms to form two *σ* bonds. The oxygen atom also has one *sp*^2^ *lp* distributed within, and one *p*_y_ lone pair distributed perpendicular to the C–O–C plane, respectively. Figure 1f,g shows the partial charge density within the energy range from –1 to 0 eV (with reference to *E*_F_) for both phases. P1 shows strong *π* orbitals surrounding carbon atoms, which is consistent with the PDOS results in Fig. 1d. In contrast, as shown in Fig. 1g, P2 exhibits a clear mixing/merging between the oxygen *sp*^2^ *lp* orbital and the carbon *π* orbitals (*lp*-*π* interaction). Based on these evidences, we believe that reducing angle *α* in P1 (thus the shorter C–C distance beneath the oxygen) promote interactions of *π* orbitals of the two carbon atoms and the empty *sp*^2*^ *lp* orbital of oxygen atom (above *E*_F_) in Fig. 1d. A charge transfer takes place from the carbon *π* orbitals to the oxygen *lp* orbital, evidenced by a shift of the oxygen *p*-electron DOS from above the *E*_F_ in P1 to below the *E*_F_ in P2 (and thus overlaps with the carbon *π* DOS peaks). Because there are no other notable differences in the DOS and the partial charge density of both phases, we believe that this *lp*–*π* interaction stabilizes the P2 phase.

The *lp*–*π* interaction is different from the well-known C–O–C ether group (with a bond angle *α* ∼110°), in which the oxygen atom exhibits *sp*^3^ hybridization. It should also be distinguished from the weak inter-molecular *lp*–*π* interaction that has attracted lots of attention in the past two decades owing to its crucial role in several essential phenomena in biological systems, such as protein folding, DNA/RNA stacking and drug–receptor interactions^44^,^45^,^46^, where the inter-molecular *lp*–*π* interaction takes place between *π*-deficient aromatic rings and lone pair-containing species. The unique *lp*–*π* interaction in C_8_O exists within a single crystal/molecule and it stems from the *π*-sufficient carbon interacting with the empty oxygen *sp*^2*^ *lp* orbital. We will term it as intra-molecular *lp*–*π* bond. Its bond energy can be approximated as the calculated energy barrier value ∼100 meV (Fig. 1b).

Figure 1c and Supplementary Fig. 3 show the relative total energy versus *a*, PDOS and partial charge density for the C_4_O. The C_4_O shares a similar crystal structure as C_8_O. The *lp*–*π* interaction is also observed in C_4_O (Supplementary Fig. 3b), which tends to stabilize the second phase (Fig. 1c). But it appears not strong enough, as there is no energy barrier to separate P1 and P2 in Fig. 1c. Nevertheless, these results confirm critical role of the *lp*–*π* interaction in GO crystals with aligned epoxy groups.

### Reversible phase transitions of C_8_O and C_4_O

Interestingly, our DFT simulations show that applying an external **E**-field and a mechanical force can lead to a reversible phase transition. In our calculations, the **E**-field is applied perpendicular to the basal plane (that is, *z* axis in Fig. 1a) with a magnitude varying from –0.5 to +0.5 eV Å^−1^, which has been demonstrated to be achievable in devices fabricated using two-dimensional graphene-based materials in experiments^47^,^48^. The structure is allowed to relax in the *x–y* plane.

**Figure 2:**
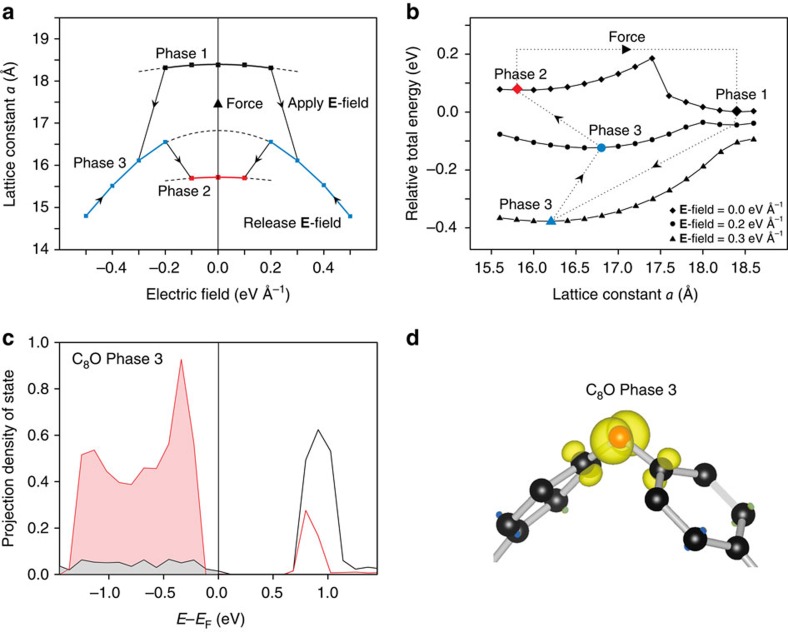
Reversible phase transition of C_8_O triggered by a combination of external E-field and mechanical force. (**a**) Lattice constant *a* as a function of the strength of an **E**-field that is applied perpendicular to the basal plane (that is, *z* axis in Fig. 1a). The black, blue and red lines represent the phase 1, phase 3 and phase 2, respectively. Black dashed lines indicate the parabolic electrostriction effect of each phase upon external **E**-field. Applying and releasing an **E**-field will cause phase transition from P1 to P2. Applying a mechanical force (stretching) will transform C_8_O back to P1. (**b**) Relative total energy as a function of lattice constant *a* under **E**-field strength of 0, 0.2 and 0.3 eV Å^−1^, respectively, to help understand the phase transition from the energetic perspective. The black, red and blue dots represent the fully relaxed crystal structures of P1, P2 and P3 upon applied **E**-field. The dotted lines with arrows show the cycle of corresponding reversible phase transition. (**c**) The PDOS results of the oxygen atoms (red) and its neighbouring carbon atoms (black) of C_8_O phase 3. The vertical lines represent the *E*_F_. Below *E*_F_, red- and grey-shaded areas indicate occupation of electrons on the p-orbitals of oxygen and carbon atoms, respectively. (**d**) Partial charge density surrounding the oxygen and its neighbouring carbon atoms within the energy range from −1.0 to 0.0 eV with reference to *E*_F_ for phase 3 C_8_O. These results are distinctive from those of phase 1 and phase 2 shown in Fig. 1 and Supplementary Fig. 2.

Figure 2a shows the change of the in-plane lattice constant *a* with varying **E**-field strength for C_8_O. The lattice constant *b* (*y* axis) exhibits a negligible change under different **E**-fields (Supplementary Fig. 4). P1 shrinks in *x*-direction under a weak **E**-field, showing a nearly parabolic relation. This could be attributed to the electrostriction effect^49^. At around 0.2 eV Å^−1^, the lattice constant *a* has a sudden drop. Beyond this point, the lattice constant *a* decreases, following another parabolic relation. A subsequent release of the **E**-field sees a gradual increase of *a* till ∼0.2 eV Å^−1^. Surprisingly, at this critical point, lattice constant *a* drops again and C_8_O transits to P2 till completely releasing the **E**-field. Applying a positive or negative **E**-field leads to the same results (Fig. 2a) because of the crystal symmetry of C_8_O. Then applying a stretching force to P2 gives rise to a phase transition back to P1, closing the loop of a reversible phase transition (Fig. 2a). Applying a stretching force to a two-dimensional material is feasible in experiments^50^.

It is a surprise to observe three parabolic curves in Fig. 2a. The top and bottom curves represent P1 and P2, respectively. The middle one appears to be the third phase. Indeed, the DOS (Supplementary Fig. 5), PDOS and partial charge density (Fig. 2c,d) distinguish it from both P1 and P2. It is thus termed as P3. The band gap of P3, ∼0.5 eV, is clearly larger than that of P1 and P2 (Supplementary Fig. 2a,b). From Fig. 2c,d, we find that there is no *π* orbital of carbon atoms just below *E*_F_ and the π* orbital is notably more profound than that of P1 and P2. Our DFT results did not show P3 under a zero **E**-field (Fig. 1b). P3 may be stable only under certain **E**-fields (Supplementary Note 2).

Through a comparison of the PDOS results of these three phases, P3 appears as a transition state from P1 to P2. The external **E**-field pushes the *E*_F_ to a lower energy level, tending to empty the *π* orbitals of P1. At the critical point (P1 transition to P3), the emptied *π* orbital would merge with the π* orbital, manifested as the more profound π* orbital in P3. With the release of **E**-field back to the critical point (P3 transition to P2), electrons would start to refill both π* orbital of carbon atoms and the oxygen *lp* orbital (because they share same energy levels in Fig. 2c), thus forming the *lp–π* bond and stabilizing the P2.

To understand the reversible transition from an energetic perspective, Fig. 2b shows the relative total energy versus *a* of C_8_O under an **E**-field strength of 0, 0.2 and 0.3 eV Å^−1^, respectively. Overall, the applied **E**-field pushes up energy of P1 with respect to another phase. Above the critical **E**-field strength of 0.2 eV Å^−1^, the energy barrier starts to disappear. The lower local minimum point at 0.2 eV Å^−1^ is P3 instead of P2. At 0.3 eV Å^−1^, the energy barrier diminishes and a spontaneously phase transition takes place as observed in Fig. 2a. Figure 2b also shows that further increasing **E**-field moves the local minimum energy point of P3 towards a smaller lattice constant, which is consistent to the reduction of *a* for P3 (Fig. 2a). Then, with releasing the **E**-field till 0.2 eV Å^−1^, the lattice constant of P3 increases (Fig. 2a). The local minimum energy points of P3 and P2 are always located to the left of the energy barrier. Re-establishment of the energy barrier as a result of releasing **E**-field will prevent C_8_O from recovering P1 but instead transit to P2.

**Figure 3:**
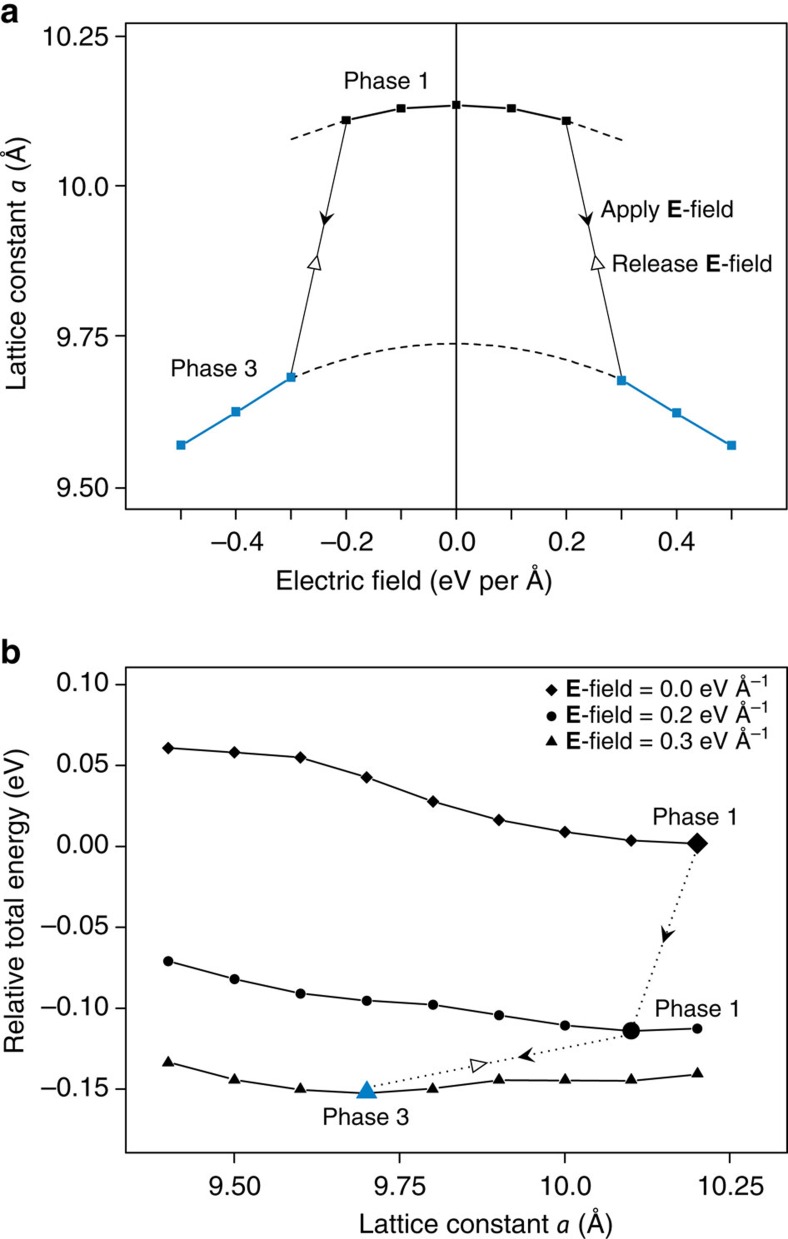
Phase transition of C_4_O triggered by E-field. (**a**) Lattice constant *a* as a function of applied **E**-field strength. The black and blue lines represent phase 1 and phase 3, respectively. Black dashed lines indicate the parabolic electrostriction effect of each phase upon external **E**-field. Applying **E**-field on P1 induces a phase transition to P3. Upon releasing the **E**-field, the C_4_O reverts to P1. (**b**) Relative total energy as a function of lattice constant *a* under **E**-field strength of 0, 0.2 and 0.3 eV Å^−1^, respectively. The black and blue dots represent the fully relaxed crystal structures of P1 and P3 upon applied **E**-field. The phase 2 is not stable, which is consistent with the result in Fig. 1c. The dotted lines with arrows show the corresponding phase transition.

Same analysis was carried out for C_4_O to further verify our understanding of the phase transition. The change of lattice constant *a* with varying **E**-field strength is shown in Fig. 3a. Figure 3b summarizes the relative total energy versus *a* under some typical **E**-field strength. Like the C_8_O (Fig. 2), C_4_O P1 gradually shrinks under a small **E**-field strength. It then undergoes a sudden phase transition to a new phase P3 upon an **E**-field greater than 0.2 eV Å^−1^. The DOS and PDOS results of C_4_O P3 (Supplementary Fig. 5c,d) are similar to those of C_8_O P3. But upon releasing the **E**-field, the C_4_O reverts to P1, owing to the lack of an energy barrier. These consistent evidences reveal common features of structural and electronic properties of these GO crystals with orderly aligned epoxy groups, and the potential of utilizing an **E**-field to trigger the phase transition.

It is clear that on-or-off state of the *lp*–*π* bond/interaction determines the reversible phase transition in C_8_O. The overall similarity of DOS/PDOS results of both phases (Supplementary Fig. 2 and Supplementary Table 1) and the localized partial charge density near the C–O–C groups in P2 (Fig. 1g) evidently indicate the local nature of this unique bond. It is thus reasonable to observe the local crystal structure changes during a phase transition. This is utterly different from SMAs and SMCs, in which the whole crystal lattice undergoes a transformation, usually resulting in distinctive crystal symmetry and electronic structures of the two phases^3^. Interestingly, the local intra-molecular *lp*–*π* bond is analogous to state-of-the-art design concept of SMPs—molecular switch,^51^ in which switching on-or-off some local inter-molecular bonds in polymer networks enables fixing of temporary shapes or recovery of the original shape. Thus, we term the *lp*–*π* bond as *lp*–*π* switch, which could provide fresh ideas in the design of SMMs at nanometre scale, benefiting from the knowledge obtained in SMP research field.

### Shape memory C_8_O

**Figure 4:**
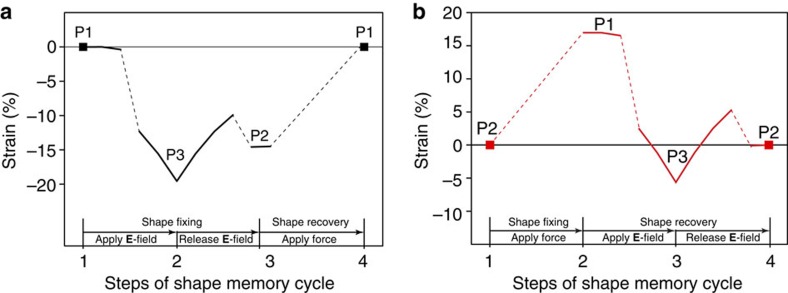
A full shape memory cycle for C_8_O. (**a**) Case 1: taking P1 as the permanent phase. An **E**-field is used as stimuli for shape fixing, where mechanical force is applied for shape recovery. The horizontal line represents zero strain with reference to P1. The maximum recoverable contraction strain is 14.5% and the maximum intermediate contraction is 19.4% that takes place at **E**-field strength of 0.5 eV Å^−1^. (**b**) Case 2: taking P2 as permanent phase. An applied stretching force is used to fix temporary shape (P1) and an **E**-field triggers the shape recovery. The horizontal line represents zero strain with reference to P2. The maximum recoverable expansion strain is ∼17%. It is worth noting that the recovery step shows a discontinuous shape change, which is a signature of the multi-SME. The dashed lines connect different phases at different steps as a guide for the shape memory cycle.

This reversible phase transition in C_8_O enables the highly desirable SME. A shape memory cycle is divided into two steps, namely shape fixing and shape recovery^19^. Figure 4 demonstrates these two steps for C_8_O. Taking P1 as the permanent shape (Fig. 4a), the **E**-field can be used as the external stimuli to fix the temporary shape (P2). The recoverable contraction strain arising from this shape-fixing step is ∼14.5%. This value is much higher than most of the SMAs (less than 8%)^3^. The shape recovery can be activated by a mechanical stretching force. In novel applications such as material-as-machine at nanometre scale, using **E**-field for temporary shape fixing is more practical in comparison with other common stimuli like mechanical force.

Most SMMs can only memorize one temporary shape in one shape memory cycle. A current research interest is to develop multi-SME that can exhibit more than one distinctive shape change in the recovery step. The resultant multi-SME provides much better controllability and more flexible shape morphing ability^51^,^52^. Thanks to its three different phases, C_8_O could have a triple-SME. As illustrated in Fig. 4b, taking P2 as the permanent shape, C_8_O can be pre-stretched and fixed to its first temporary shape P1 using a mechanical force. In the recovery step, applying and releasing an **E**-field will observe a lattice reduction, then an expansion and finally a reduction to the final permanent shape. Such a discontinuous shape change is recognized as signature of the multi-SME^51^. The triple-SME C_8_O could enable rich design possibilities in practices.

**Figure 5:**
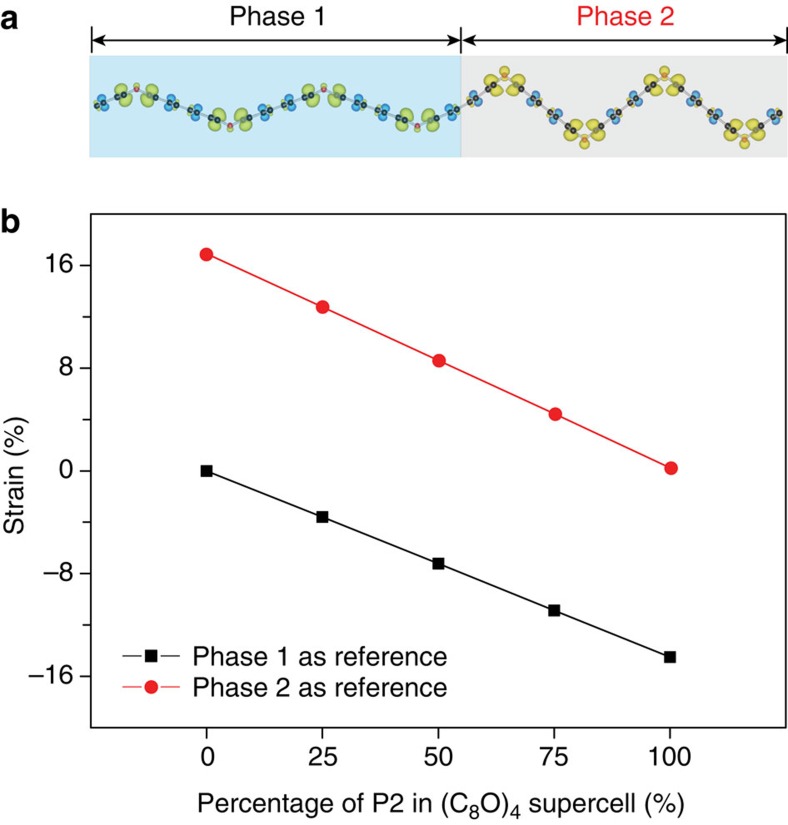
Programmability demonstrated by the stable co-existence of P1 and P2 mixture in one supercell. (**a**) A stable four-unit supercell in DFT simulations that includes two units of P1 and two units of P2. The signature partial charge density of the local *lp*–*π* bonds in P2 can be clearly seen. Other P1/P2 mixing cases in this four-unit supercell are presented in Supplementary Fig. 6. (**b**) The recoverable strain results of all possible temporary shapes from a four-unit supercell via programming the percentage of P2 (Supplementary Table 2a). The strain value is calculated corresponding to the two cases represented in Fig. 4, where the first case takes P1 as the permanent shape and the second case takes P2 as the permanent shape.

The most important feature to distinguish SMMs from other stimuli-responsive shape change material is the programmability^51^. In other words, one piece of SMM can be programmed/fixed into different temporary shapes. The co-existence of different phases and variants in one SMM is the key to achieve programmability, because different types and ratio of combinations allow tailoring the microstructures for multiple temporary shapes. On this wise, the co-existence of both P1 and P2 were carefully examined in our DFT simulations. Several supercells were created to incorporate different numbers and distribution patterns of P1 and P2 (Fig. 5 and Supplementary Fig. 6). As an example, Fig. 5a illustrates the supercell including two units of P1 and two units of P2 (2/2). Our DFT calculations conclude stability of all these coherently mixed P1/P2 phases. The signature partial charge density of *lp*–*π* bonds can be observed in the supercells, further confirming its local nature. The stability of various P1/P2 mixing can be attributed to this local chemical bond.

We propose that in applications at nanoscale, an STM or AFM tip can be employed to apply a local **E**-field or local point force to tune different mixtures of P1/P2 for the desirable temporary shapes. As a simple illustration, Fig. 5b and Supplementary Table 2a summarize the lattice constants of all possible temporary shapes of a supercell including four unit cells of C_8_O (different P2 percentage) and the programmable recoverable strain results. Larger supercells including six unit cells of C_8_O are also examined and results are summarized in Supplementary Fig. 7 and Supplementary Table 2b. All these evidences confirm the programmability of C_8_O SMM.

The maximum elastic strain and specific modulus (ratio of elastic modulus over mass density) are often chosen as the key parameters to assess the mechanical properties of materials. Leng *et al*. surveyed available SMMs in a review article^19^. Generally, a SMM with a large maximum recoverable strain usually had a low specific modulus and thus concluded an upper bound (Supplementary Fig. 8). At its maximum recoverable strain of 14.5%, our C_8_O has a specific modulus about ten times higher than the upper bound. Given the same specific modulus 4.3 GPa cm^3^ g^−1^, C_8_O has a maximum recoverable strain approximately threefold larger than the upper bound. Another important practical consideration is the actuation frequency. Commonly used thermal-responsive SMMs exhibit small actuation frequencies ∼1 Hz, which is recognized as a bottleneck, severely restricting their applications. The required external stimuli of C_8_O SMM (that is, **E**-field and mechanical force) should, in principle, allow much higher actuation frequencies.

**Figure 6:**
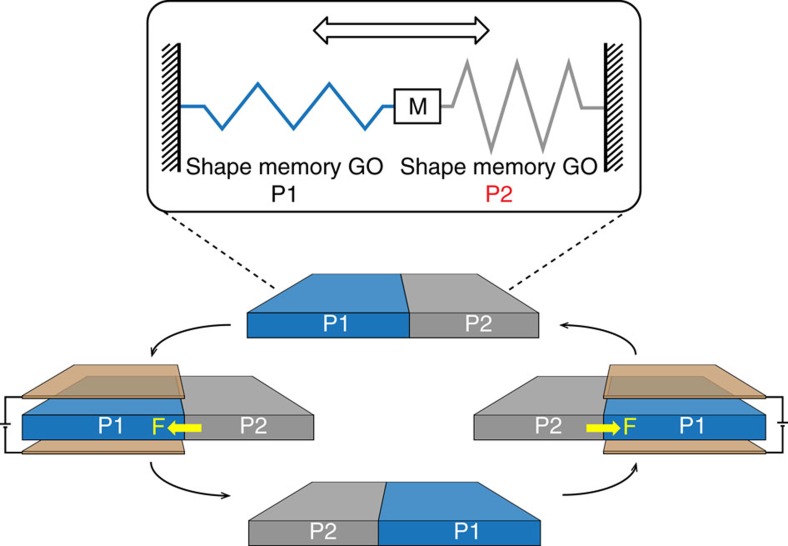
A simple prototype design of a two-way actuator using shape memory C_8_O. The actuator includes one mass element (M) and two segments of shape memory C_8_O. The two phases P1 and P2 are indicated as blue and grey region, respectively. Applying an **E**-field on the P1 segment will cause a phase transition to P2, generating a shrinkage deformation and thus a stretching force to the P2 segment. As long as the force is large enough (for example, 0.5 eV Å^−1^), a simultaneous phase transition from P2 to P1 will take place (Supplementary Fig. 9). Element M in the middle may move back and forth if applying the **E**-field alternatively on either segment, as indicated by the arrow.

At the end of this section, we propose a simple prototype design of two-way actuator based on C_8_O SMM (Fig. 6). In this design, two segments of C_8_O (one in P1 and the other one in P2) are connected to the opposite sides of a mass element M, whereas the other end is fixed. Applying a local **E**-field to the P1 C_8_O (left segment in Fig. 6) will cause it to contract and consequently generate a stretching force **F** on M and then to the P2 C_8_O (right segment in Fig. 6). As long as the applied **E**-field strength is sufficiently high, for example, 0.5 eV Å^−1^ (Supplementary Fig. 9), the generated force can transform the right segment from P2 to P1. As a result, phases of the left and right segments swap as illustrated in Fig. 6. Applying the **E**-field alternatively on either segment can generate a repeated back and forth linear motion of element M (Supplementary Note 3). A clear advantage of this design is that only an **E**-field is required to trigger the shape memory cycle and operate this actuator, which is feasible and highly desirable for designs of nano-mechanical devices.

## Discussion

From a practical perspective, it is imperative to discuss the finite temperature effects. Using molecular dynamics simulations, Huang *et al*. simulated the thermal fluctuation of a C_8_O structure with linear alignment of epoxy groups on single side of graphene layer at 1,500 K and concluded no structural destruction^35^. Our C_8_O has a lower total energy than that in Huang's study. It should have good structural stability at room temperature. In addition, thermal fluctuation causes out-of-plane distortion of a suspended two-dimensional materials, which could compromise the control of in-plane strain in applications of our SMM C_8_O. This effect can be controlled by appropriate designs. Xu *et al*. reported that thermally excited out-of-plane distortion of a suspended graphene or chemically functionalized graphene depended on its length-to-width ratio. At room temperature, for a graphene layer with an edge length of 50 nm and an aspect ratio of one, the out-of-plane displacement is on the order of 2 Å (ref. 53) In Huang's simulations, thermally induced out-of-plane ripple in a C_8_O periodic supercell (aspect ratio close to 1 and supercell edge length approximately 41 Å) was around 2.5 Å at 1500 K (ref. 35), from which the distortion can be estimated as 1 Å at 300 K. Fasolino *et al*. used molecular dynamics simulations to study the intrinsic thermally excited ripples in a sufficiently large graphene supercell and found a ripple magnitude of 0.7 Å and a wave length of ∼80 Å (ref. 54). These out-of-plane displacements are small in comparison with the lateral size or ripple wave length. Thus, control of the aspect ratio is a very convenient means to suppress this distortion. Another option is to use a substrate, which will be discussed later.

To examine the structure and phase stability of our C_8_O SMM in atmosphere, four types of gas molecules, O_2_, CO_2_, H_2_O and N_2_, were added in the supercells of P2 C_8_O. The P2 C_8_O was selected because its weak *lp*–*π* bond could be affected by the gas molecules. The crystal structure and partial charge density results in Supplementary Fig. 10a–d demonstrate negligible effects. We, therefore, expect that the SME remains valid in atmosphere. Nevertheless, despite the negligible effect of water molecules on the *lp*–*π* bonds, the hydrogen-rich or humid environment should be avoided because hydrogen can transform epoxy groups to hydroxyl groups^32^,^34^.

A substrate is required in many applications. Supplementary Fig. 11 shows the equilibrium crystal structure and partial charge density results of P1 and P2 on top of a graphene nano-ribbon substrate. In comparison with Fig. 1, there are no notable differences. In addition, our DFT results show that the energetic order of these two structures is swapped by applying an external **E**-field of 0.3 eV Å^−1^. The **E**-field induced phase transition, therefore, should take place (Supplementary Note 4).

The bi-layered structures were also investigated in our DFT simulations. Bi-stable phases are observed (Supplementary Fig. 12). The P1 crystal structure resembles the monolayer case. Interestingly, two types of C–O–C angles, 130.3° and 106.3°, are distributed alternatively along the *x* axis in the metastable phase. Together with the partial charge analysis, we can conclude this metastable phase consisting of both P1 and P2 components of the monolayer C_8_O. The total energy difference of these two phases (128 meV) is close to the monolayer C_8_O (76 meV). These evidences suggest that **E**-field and stress could lead to phase transition and thus the SME in bi-layered C_8_O.

Next, we consider the influence of some typical defects. First, previous studies suggested that a mixing pattern of C_8_O and C_4_O could exist^35^,^41^. A supercell was then constructed to include one unit cell of C_8_O and C_4_O in *x* axis direction (Supplementary Fig. 13). Our DFT calculations identified two stable structures. We also found that tuning the **E**-field could change the energetic order of these two structures, suggesting a phase transition triggered by **E**-field.

Regarding the epoxy lines, there are two common defects: oxygen vacancy and redundant oxygen adatom. These two types of defects were produced in supercells of perfect P1 and P2 phases of a size of 1 × 5, 1 × 7 and 1 × 9, respectively, to account for different defect concentrations (20%, 15% and 11%, respectively). The oxygen vacancy was generated by removing one epoxy group from the supercells (Supplementary Fig. 14). The redundant oxygen adatom was introduced in the supercells to form either epoxy pair or carboxyl pair (Supplementary Fig. 15). These defects cause local structural distortions only up to the first nearest-neighbour epoxy group. The P1 and P2 supercells remain stable as long as the defect concentration is lower than 20% (or epoxy line is longer than 1.2375, nm). The signature *lp*–*π* bonds appear in partial charge density of the P2 supercells (beyond the first nearest neighbour of defects). Supplementary Note 5 provides detailed descriptions. We can expect that at a reasonable defect density (for example, smaller than 20%), these defects should have negligible influences on the SME of our C_8_O SMM.

In the following, we will discuss possible fabrication routes of C_8_O SMM in experiments. The first step is to achieve homogeneous functionalization of epoxy groups on graphene. The widely used Hummers method usually leads to chemical inhomogeneity of oxygen functional groups, making this choice unfavourable. There are two promising alternative ways. Recently, Hossain *et al*. reported that oxidation of epitaxial graphene using atomic oxygen in ultrahigh-vacuum produced uniform epoxy functionalization^36^. Another route is to use vacuum thermal annealing process to remove functional groups other than epoxy^33^.

The next step is to realize the linear alignment of epoxy groups. This topic has attracted many studies in the past for the purpose of unzipping graphene layers^38^,^39^,^55^,^56^,^57^. DFT simulations indicated that linear alignment could effectively reduce strains in a graphene layer caused by the epoxy groups. Huang *et al*. employed DFT calculations and cluster expansion approach to explore a configuration space including ∼17,000 different GO structures. Interestingly, they concluded that a C_8_O crystal structure with well-ordered linear pattern of epoxy group is the ground-state structure provided that graphene is oxidized in one side^35^. These theoretical studies provide a solid thermodynamic ground for fabricating GO with well-ordered epoxy lines. In their study of growth mechanisms, Sun *et al*. found that the formation of an epoxy linear trimer would attract and trap nearby oxygen adatom to diffuse to the end of trimmer, leading to the growth of an epoxy line^55^. To guide growth direction, there are several options. One possibility is to apply uniaxial strain in graphene layer to break symmetry of the honeycomb structure. This can be done by placing a pristine graphene on a flexible substrate and then either stretching or bending the substrate. Ma *et al*. found that the uniaxial strain can reduce the reaction energy barrier to promote growth of epoxy lines along certain directions^57^. The second option is to generate periodic rippling in graphene layers via mechanical compression. Residual strain on the ripple ridges would make the C–C bonds more reactive to oxygen adatoms to form the linear epoxy chains. Our recent theoretical study showed that the rippling wavelength could be controlled down to 1 nm range, which can match the periodicity of C_8_O (ref. 58). The third option is to take advantage of the experimental techniques from Hossain *et al*., in which STM tips were used to induce desorption of chemisorbed oxygen, move oxygen adatoms and thus pattern the epoxy groups at atomic precision on graphene layers^36^. It is plausible to use STM tips to pattern parallel epoxy trimmers and subsequent oxidation will lead to spontaneous growth of epoxy lines. All three options can be combined with the vacuum oxidation setup from Hossian *et al*. The first and second option could be combined with the vacuum annealing method from Mattson *et al*.^33^.

Note that in these previous studies^38^,^39^,^55^,^56^,^57^, some further steps were required to unzip the GO with linear epoxy chains into nano-ribbons. For instance, Fujii's experiment used an AFM tip to press the oxidized graphene layers and the resultant local stress led to the structural rupture. Ma *et al*. suggested that further oxidization of the epoxy chains would lead to unzipping^57^. Clearly, these extra experimental steps are not needed for our case.

In summary, this paper reports a two-dimensional shape memory C_8_O triggered by **E**-field and mechanical force. A unique chemical bond, *lp*–*π* interaction, is revealed as the physical origin of the SME. Besides traditional single SME, an interesting triple SME is also achievable. The overall mechanical properties of the shape memory C_8_O are significantly better than those of currently available SMMs. In addition, the **E**-field and mechanical force stimuli could overcome the limitations (such as, actuation frequency only about 1 Hz) of commonly employed thermal stimuli at small size scales.

## Methods

The Vienna *ab initio* simulation package (VASP v.5.3.3) was used to perform DFT calculations in this study. Projector augments wave method and the generalized gradient approximation were employed^59^,^60^. A plane-wave cutoff energy was set as 800 eV. A Monkhorst–Pack gamma-centred *k*-points mesh of 42 × 6 × 1 was adopted for C_8_O unit cell (Fig. 1a). A similar k point mesh density was adopted for other supercells. As periodic boundary conditions were employed in VASP, thick vacuum layers were included to minimize interlayer interactions. An interlayer spacing of 20 Å was used throughout, which represents a good balance between computational accuracy and efforts. To hold this interlayer space constant, the VASP source code (constr_cell_relax.F) was modified to allow the cells to relax within the basal plane only. In all cases, the atomic positions were allowed to relax in all directions. Before being subjected to an external **E**-field, all structures were fully relaxed to determine their equilibrium lattice constants. The relative change of in-plane lattice constants under an applied **E**-field with respect to the equilibrium values were defined as deformation strain. The dipole and quadrupole corrections were adopted for all our simulations. We used VASP and Phonopy-1.10 to calculate phonon frequency^43^.

### Data availability

The data that support the findings of this study are available from the corresponding author upon request.

## Additional information

**How to cite this article**: Chang, Z. *et al*. Two-dimensional shape memory graphene oxide. *Nat. Commun.* 7:11972 doi: 10.1038/ncomms11972 (2016).

## Supplementary Material

Supplementary Information Supplementary Figures 1-15, Supplementary Tables 1-2 and Supplementary Notes 1-5 and Supplementary References
