# Supplementary material for: Two-dimensional shape memory graphene oxide
Source: Nat Commun. 2016 Jun 21;7:11972. doi: 10.1038/ncomms11972 (PMC4919543; doi:10.1038/ncomms11972)
Supplement: Supplementary Information — Supplementary Figures 1-15, Supplementary Tables 1-2 and Supplementary Notes 1-5 and Supplementary References [file ncomms11972-s1.pdf]

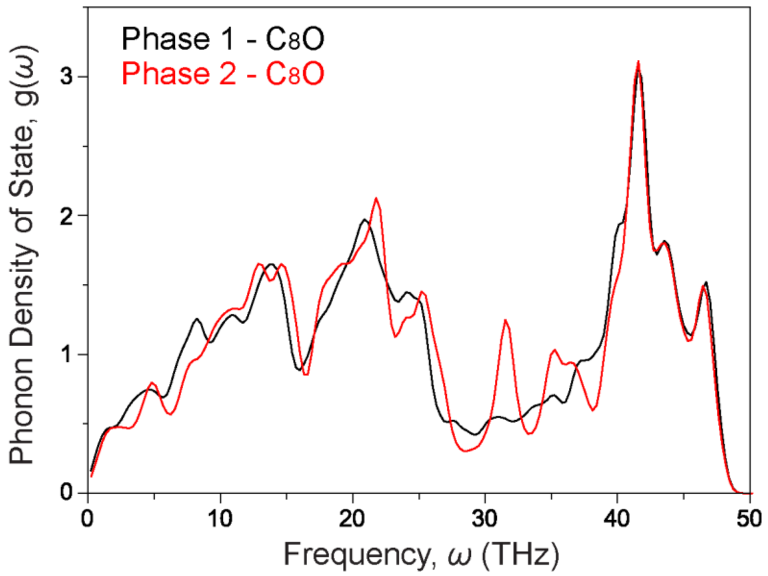

Supplementary Figure 1: Phonon density of states ($g(\omega)$) *vs.* frequency(ω). As indicated, the black and red lines represent for P1 and P2-C_8O GO. Based on Supplementary Equation 1 shown below,[1] the calculated phonon free energy using harmonic approximation is -0.836 and -0.816 eV, respectively.

$$E_{\mathrm{free}} = Nk_B T\int_0^{\omega_{\max}} \ln\left[2\sinh\left(\frac{\hbar\omega}{2k_B T}\right)\right] g(\omega)d\omega \qquad \text{Supplementary Equation 1}$$

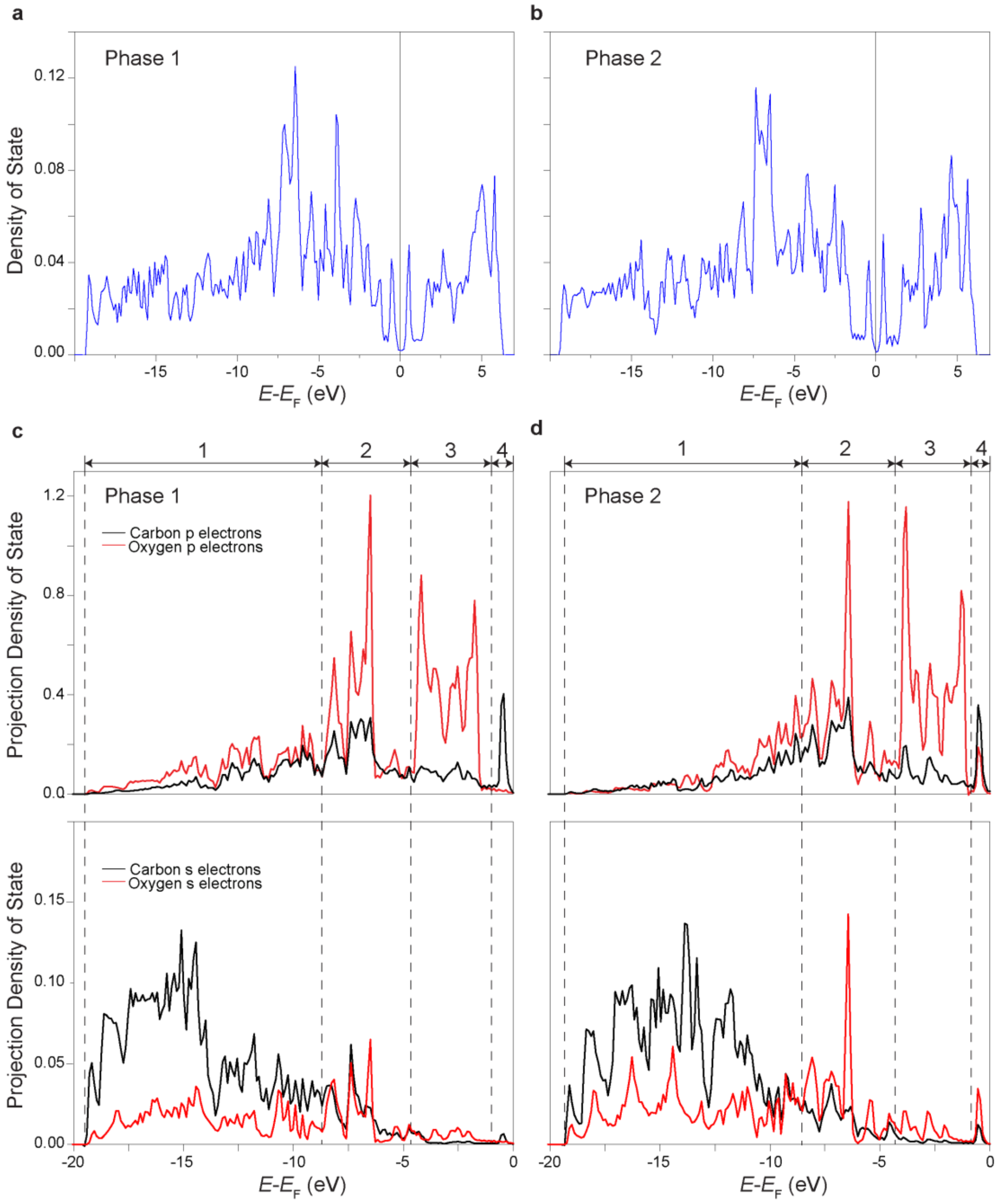

Supplementary Figure 2: The density of state (DOS) and projection density of state (PDOS) for bi-stable phases of C_8O. (a, b) DOS of Phase 1 and Phase 2, respectively. (c, d) The *p*-electrons and *s*-electrons PDOS of oxygen atom (red) and its neighbouring carbon atoms (black) of Phase 1 and Phase 2, respectively. The energy levels are divided in to four regions (separated by the dashed lines) with reference to E_F, indicated as 1, 2, 3 and 4 in (c, d). The partial charge density of these four regions is shown in Supplementary Table 1.

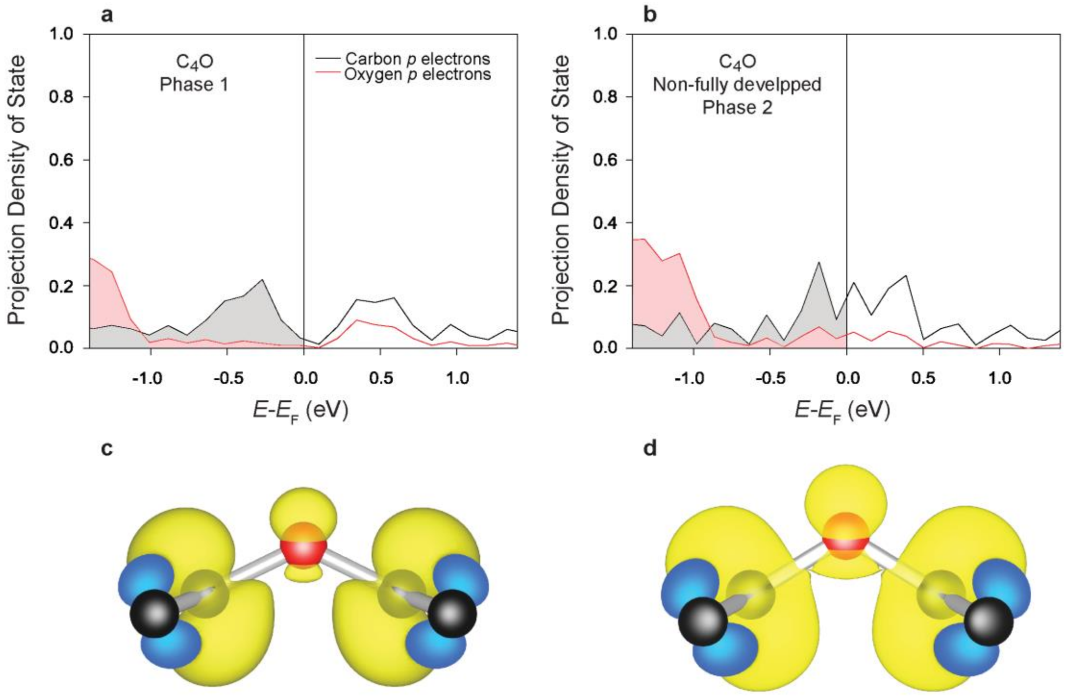

Supplementary Figure 3: The PDOS and partial charge density distribution of C_4O crystal. (a) The *p*-electrons of oxygen atom (red) and its neighbouring carbon atoms (black) of Phase 1. (b) The *p*-electrons of oxygen atom (red) and its neighbouring carbon atoms (black) of non-fully developed Phase 2. The vertical lines represent the Fermi level (E_F). Below E_F, red and grey shaded areas indicate occupation of electrons on the *p*-orbitals of oxygen and carbon atoms, respectively. (c) and (d) Partial charge density of the corresponding PDOS within the energy range from -1.0 to 0.0 eV with reference to E_F.

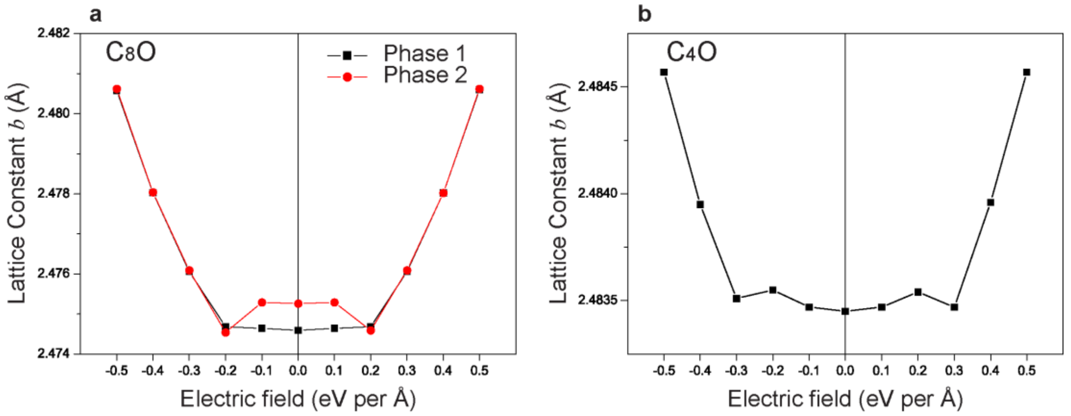

Supplementary Figure 4: Lattice constant *b* as a function of the applied E-field for C_8O and C_4O. (a) C_8O: The black line is for Phase 1 and the red line is for Phase 2. The calculated maximum strain of lattice constant *b* is 0.242% at **E**-field of 0.5 eV per Å. (b) C_4O: The calculated maximum strain of lattice constant *b* is 0.045% at **E**-field of 0.5 eV per Å.

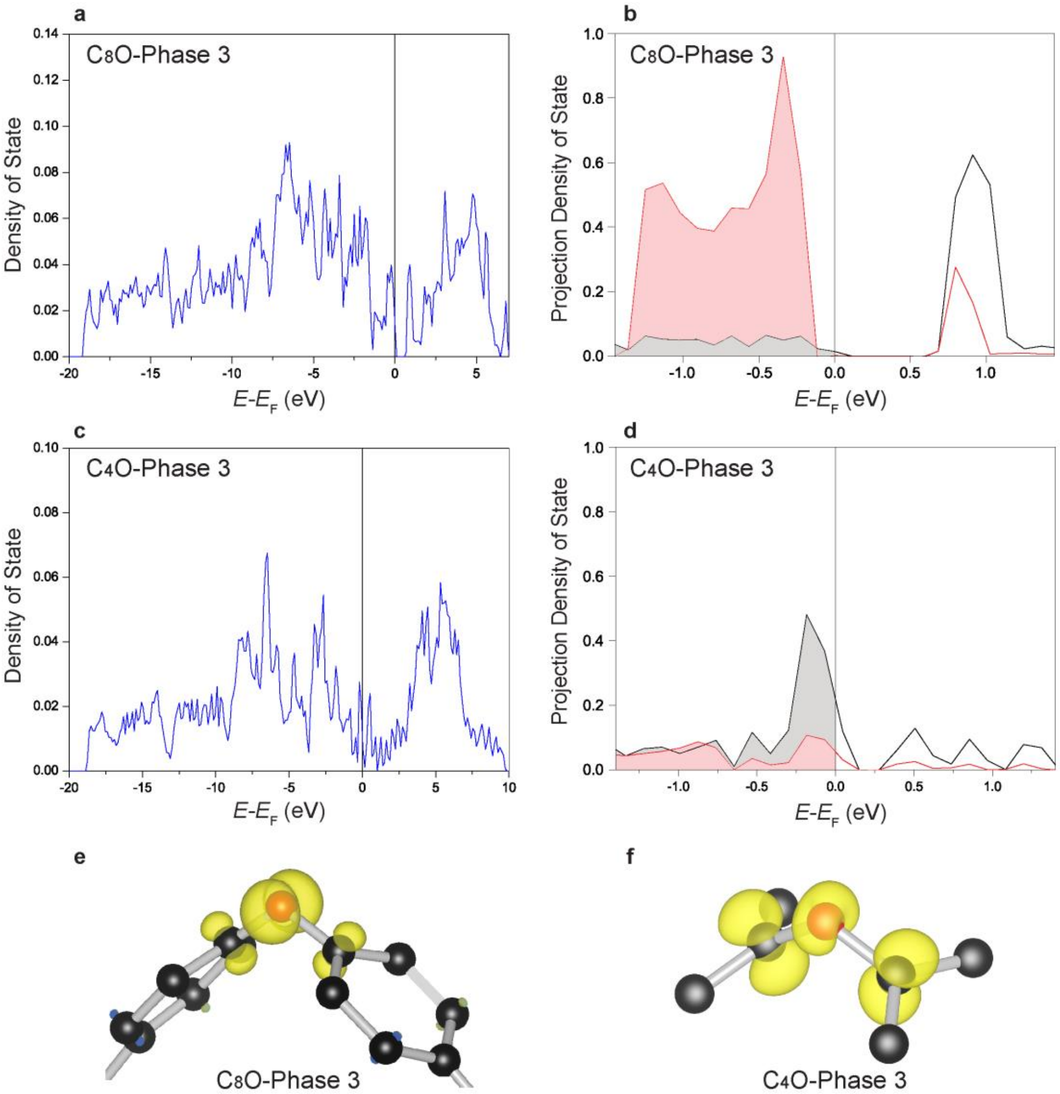

Supplementary Figure 5: Evidences of Phase 3 for C_8O and C_4O at E-field of 0.3 eV per Å (a) The DOS results of C_8O Phase 3. (b) The PDOS results of the oxygen atoms (red) and its neighbouring carbon atoms (black) of C_8O Phase 3. (c) The DOS results of C_4O Phase 3. (d) The PDOS results of the oxygen atom (red) and its neighbouring carbon atoms (black) of C_4O Phase 3. The vertical lines represent E_F. Below E_F, red and grey shaded areas indicate occupation of electrons on the *p*-orbitals of oxygen and carbon atoms, respectively. (e) and (f) Partial charge density surrounding the oxygen and its neighbouring carbon atoms within the energy range from -1.0 to 0.0 eV with reference to E_F, for C_8O Phase 3 and C_4O Phase 3, respectively. These results are distinctive from those of Phase 1 and Phase 2 shown in Fig. 1, Supplementary Figure 2 and Supplementary Figure 3.

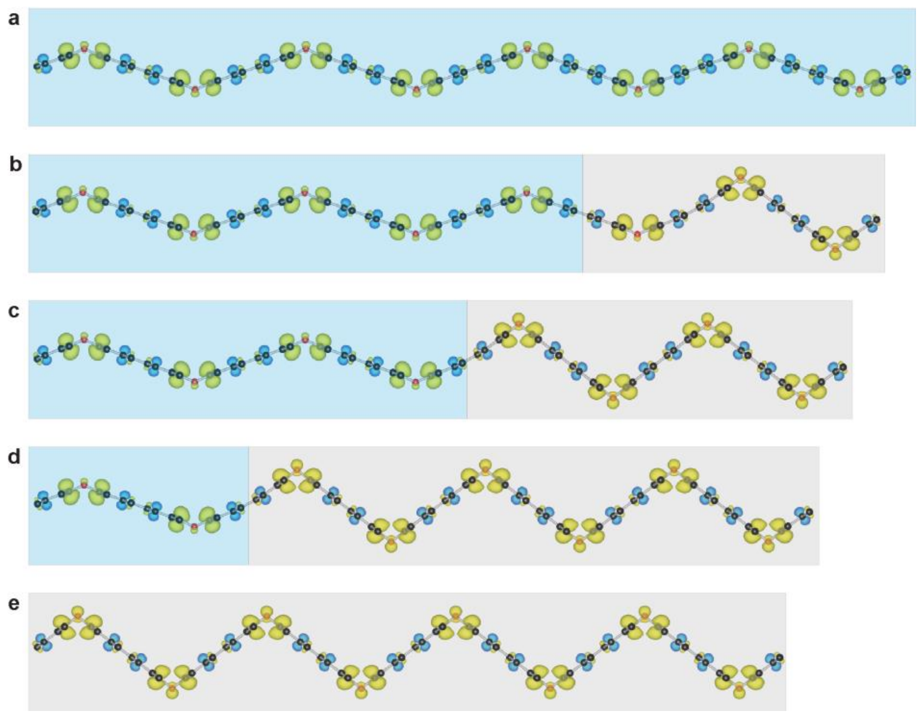

Supplementary Figure 6: Equilibrium atomistic structures of $(C_8O)_4$ supercells including different types of phase mixtures. The Blue shaded region represents Phase 1 and grey shaded regions represent Phase 2. The P1:P2 ratios from (a) to (e) are 4:0, 3:1, 2:2, 1:3 and 0:4, respectively. The atomistic structures have been fully relaxed in DFT simulations. These results conclude that Phase 1 and Phase 2 can stably co-existed in a single crystal. The equilibrium constants are summarised in Supplementary Table 2.

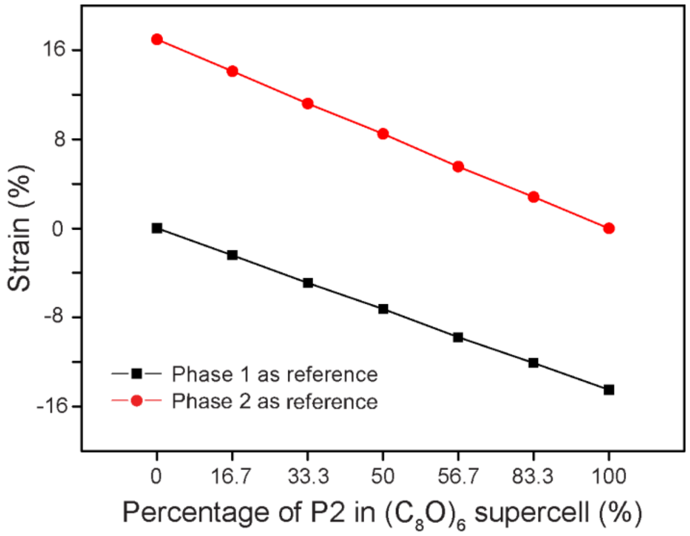

Supplementary Figure 7: Recoverable strain outputs arising from programming the percentage of Phase 2 in $(C_8O)_6$ six-unit supercells. A maximum in-plane strain of -14.5% can be obtained if set the supercell with pure Phase 1 as the reference state (black line). If taken the supercell with pure Phase 2 as a reference (red line), a maximum in-plane strain of 17% can be obtained. Although the maximum recoverable strain is identical to the four-unit supercells, a larger supercell can be programmed into more temporary shapes and thus more recoverable strain outputs.

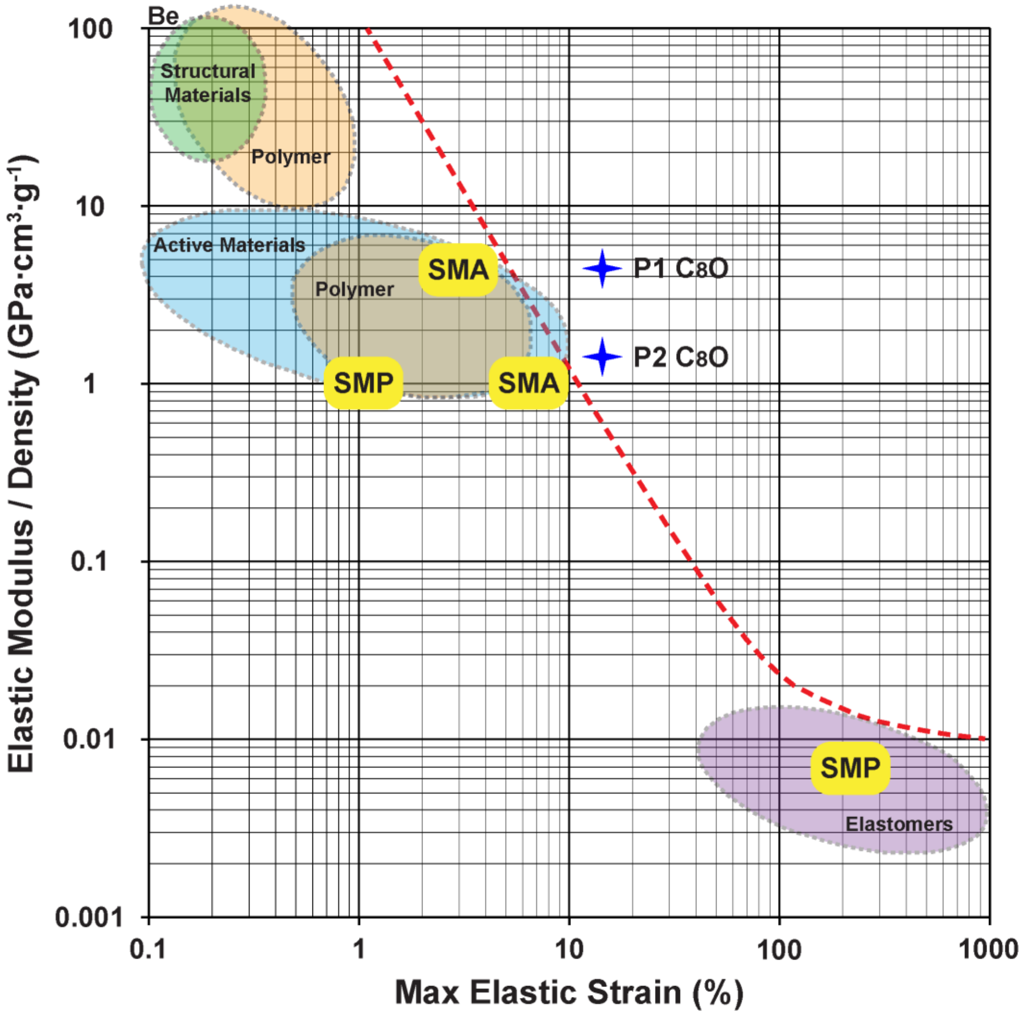

Supplementary Figure 8: A comparison of actuation performance between conventional actuation materials and two-dimensional shape memory C_8O. The specific modulus (ratio of elastic modulus over density) and maximum recoverable elastic strain are two key performance parameters. The performances of common materials (including SMMs highlighted in yellow) are adapted from Supplementary Ref. 2 and 3. An actuation material with a high maximum elastic strain usually has a small specific modulus. The red dashed line denotes the upper bound for conventional actuation materials. Two major shape memory materials, shape memory alloy and shape memory polymer, are noted as SMA and SMP in this figure, respectively. Martensite-austensite phase transformation is the underlying mechanism for SMA and shape memory effect of SMP is originate from glass transition between a hard to a soft phase. Blue stars represent the Phase 1 and Phase 2 of the shape memory C_8O. The calculated recoverable strain and specific modulus is 14.5% and 4.3 $GPa \cdot cm^3 \cdot g^{-1}$, respectively.

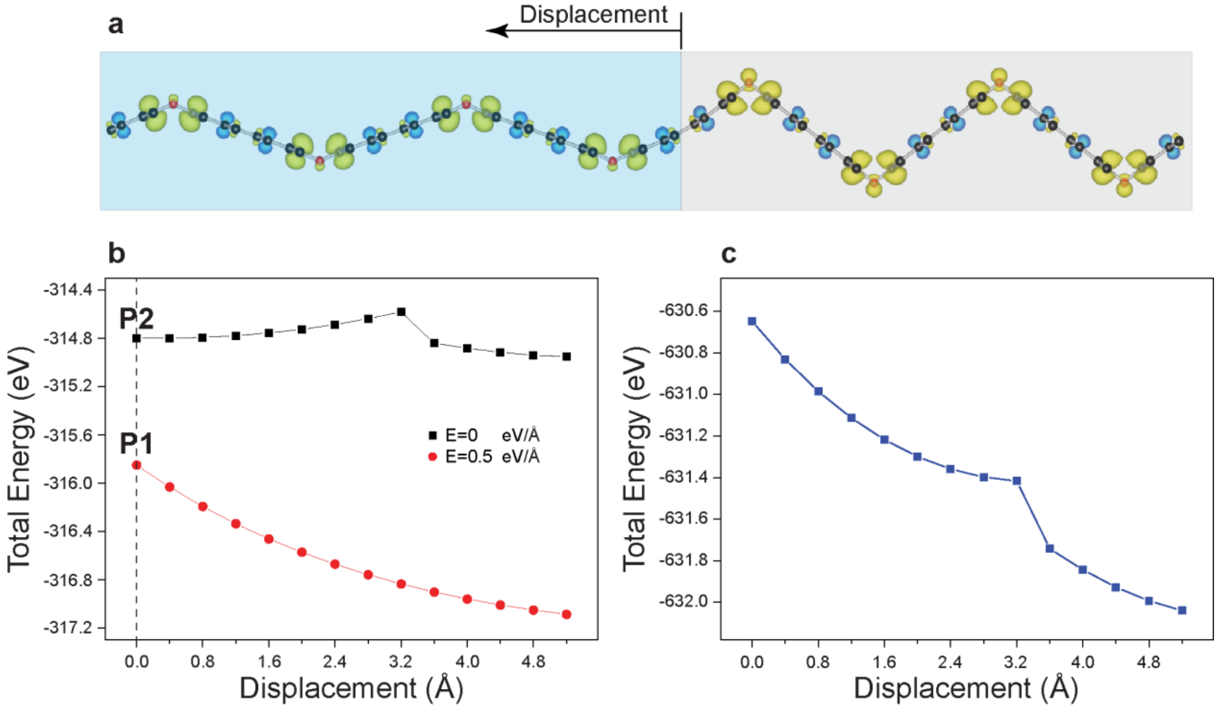

Supplementary Figure 9: Total energy *vs.* displacement of the mid-point for a $(C_8O)_4$ supercell with P1/P2 ratio of 2:2. (a) Atomistic structure of the $(C_8O)_4$ four-unit supercell. The mid-point is located at the interface between P1 and P2. Displacement of the mid-point is labelled. (b) Total energy of the P2 segment (right) and P1 segment (left) as a function of displacement (d) under a zero **E**-field and an **E**-field strength of 0.5 eV per Å, respectively. The dashed line represents zero displacement. (c) Total energy of the whole system as a function of d when the P1 segment (left) is being subject to a local external **E**-field strength of 0.5 eV per Å. This result is obtained by summarizing the two curves in (b).

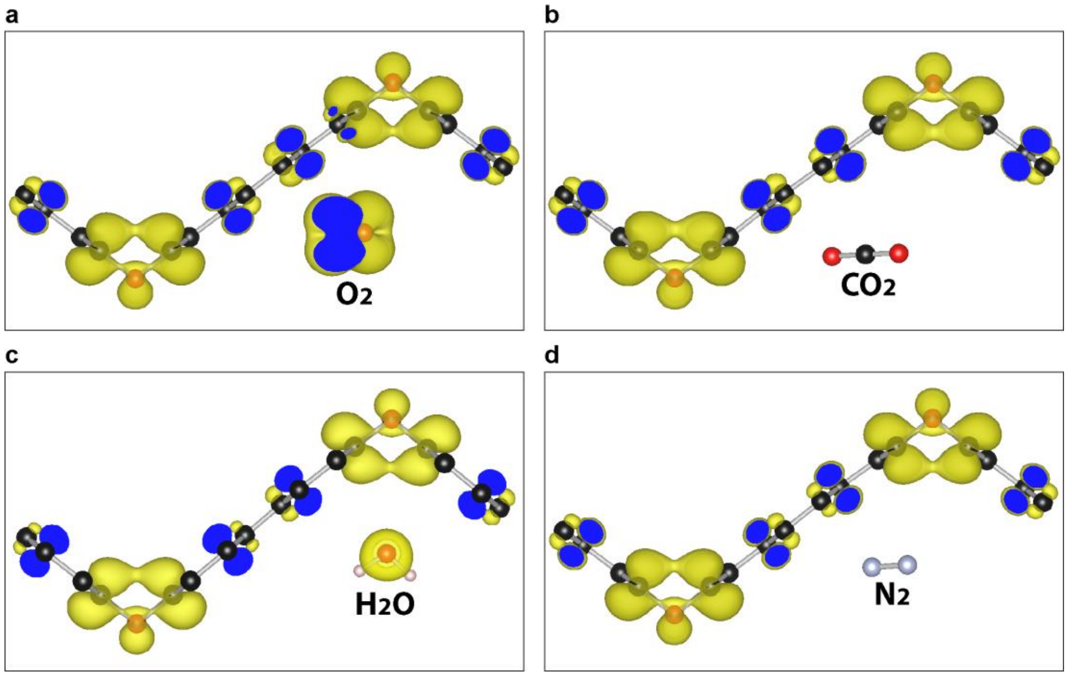

Supplementary Figure 10: Examining the influence of atmosphere. (a) – (d) Fully relaxed P2 C_8O supercells with oxygen, carbon dioxide, water and nitrogen molecules added. Partial charge density

results (within the energy range from -1.0 to 0.0 eV with reference to E_F) show intact *lp*-*π* bonds. This suggests that these molecules have nearly no effect on the phase stability of P2 C_8O.

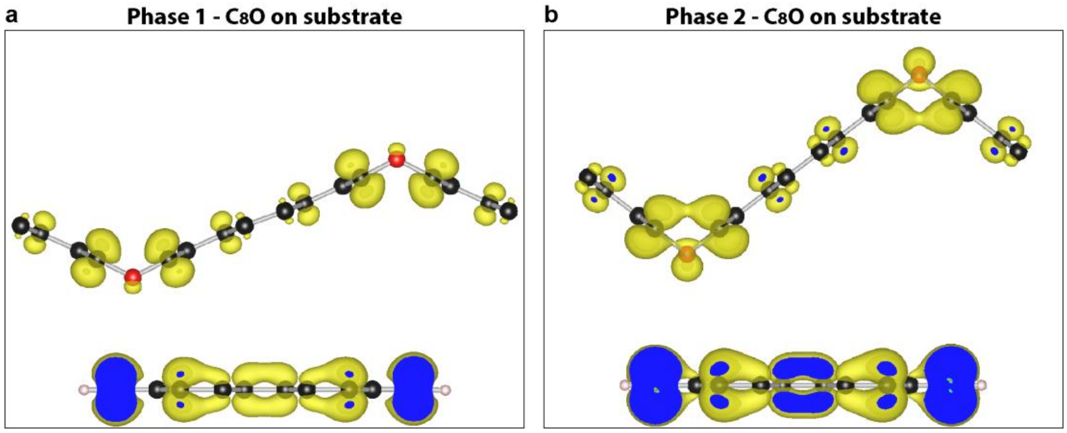

Supplementary Figure 11: Examining influences of a substrate. A layer of graphene nanoribbon was placed underneath C8O GO crystals as a substrate in this simulation. Its edges were terminated by hydrogen atoms. Crystal structure and partial charge density (within the energy range from -1.0 to 0.0 eV with reference to E_F) of fully relaxed P1-C8O (a) and P2-C8O (b) with an identical graphene substrate. The calculated total energy of P1/P2-C8O GO crystals on graphene substrate are -250.359 and -250.350 eV, respectively. Under an **E**-field of 0.3 eV per Å, the total energy values change to -250.428 and -250.626 eV, respectively. The P2-C8O crystal on substrate becomes energetically more stable (phase transformation can be triggered even C8O GO crystals are transformed onto a substrate). The swap of energetic order caused by **E**-field suggests the P1 to P2 phase transition should take place.

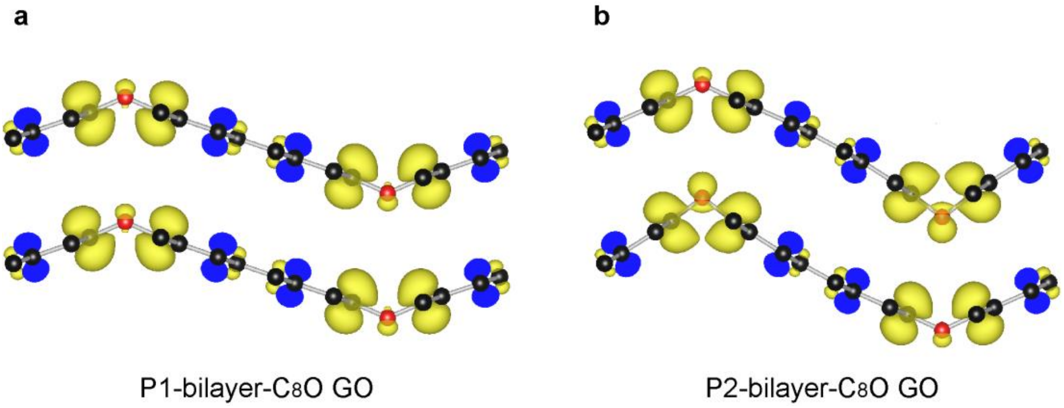

Supplementary Figure 12: Bi-stable phases of bilayer C_8O GO. Partial charge density of fully relaxed P1-bilayer- C8O (a) and P2-bilayer- C8O GO (b) within the energy range from -1.0 to 0.0 eV with reference to E_F. The lattice constant *a* is found to be 18.4 and 17.2Å, respectively. The calculated total energy of these two stable phases are -314.977 and -314.849 eV (that is total energy difference of 128 meV). The maximum recoverable strain is about 6.5%.

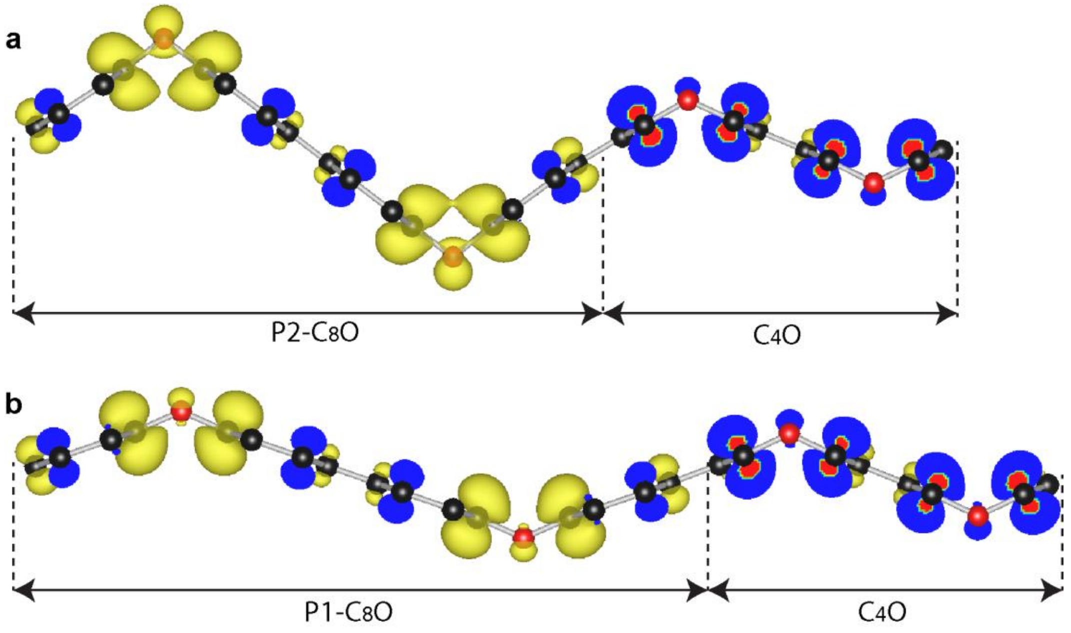

Supplementary Figure 13: Stable co-existence of C_4O and C_8O GO mixtures. Structural configurations and partial charge density of fully relaxed phase 2 (a) and phase 1 (b) of C_8O GO mixed with C_4O GO within the energy range from -1.0 to 0.0 eV with reference to E_F. The mixture ratio is 1:1 (*i.e.* P1-C_8O/C_4O and P2-C_8O/C_4O). The C-O-C angle and partial charge density results indicate that the first structure consist of one C_8O P1 unit and one C_4O P1 unit. The second structure consists of one C_8O P2 unit and one C_4O P1 unit. P1-C_8O/C_4O crystal is more stable at zero **E**-field, the calculated total energy of P1 and P2-C_8O/C_4O crystal is -241.002 and -240.867 eV, respectively. At **E**-field of 0.3 eV per Å, the corresponding total energy values are -241.044 and -241.108 and eV, which suggests that P2-C_8O/C_4O crystal becomes energetically more stable (that is phase transformation from P1 to P2) upon an appropriate external electric field.

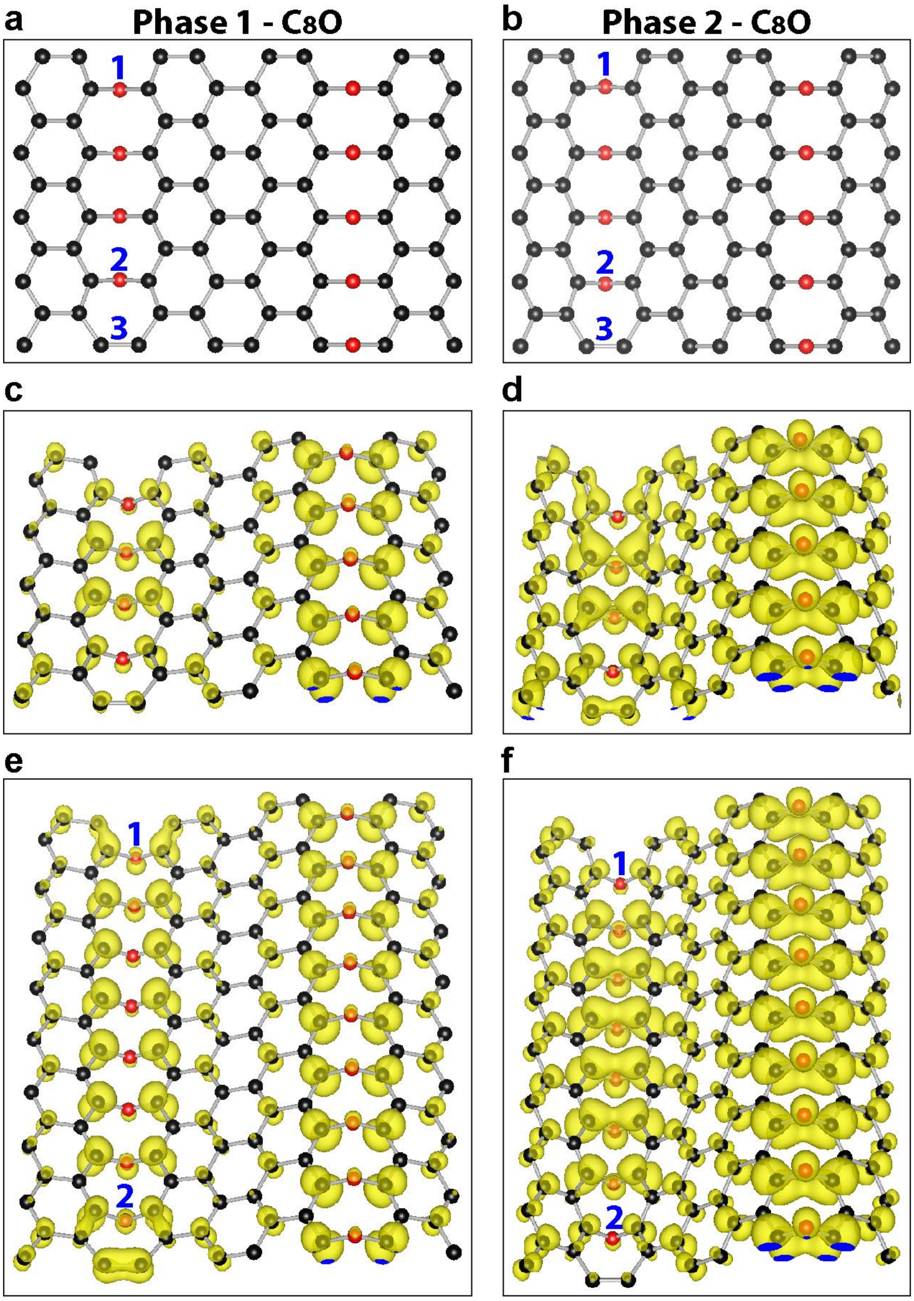

Supplementary Figure 14: Phase 1 and phase 2 C_8O GO supercells with oxygen vacancy defects by removing one oxygen atom from the epoxy line. One oxygen atom is removed at site 3 in the P1 supercells (a) and P2 supercells (b). The perfect epoxy line length is measured by the length between site 1 and site 2. Note that the lattice constant a for perfect P1&P2-C_8O GO is 18.384 and 15.718 Å. (c) and (e) Partial charge density results of P1-C_8O 1x5 and 1x9 supercells (within the energy range from -1.0 to 0.0 eV with reference to E_F), respectively. It appears like that a sp^2 carbon-carbon bond forms at site 3 upon removal of the oxygen atom (i.e. the defect rate of 20% and 11%). The fully relaxed lattice constant a is 17.934 and 18.223 Å, respectively. (d) and (f) Partial charge density results of P2 1x5 and 1x9 supercells (within the energy range from -1.0 to 0.0 eV with reference to E_F), respectively. The signature *lp-π* orbital charge density can be seen beyond the first nearest neighbour of defect. The fully relaxed lattice constant a is 15.374 and 15.510 Å, respectively. The crystal structure and partial charge density results indicate that this defect only affect the structure locally and it almost has no effects as long as concentration lower than 20% (the length of perfect epoxy line equals or

larger than 9.9 Å). The length of perfect epoxy line is measured to be 9.9 or 17.723 Å for these two different size supercells.

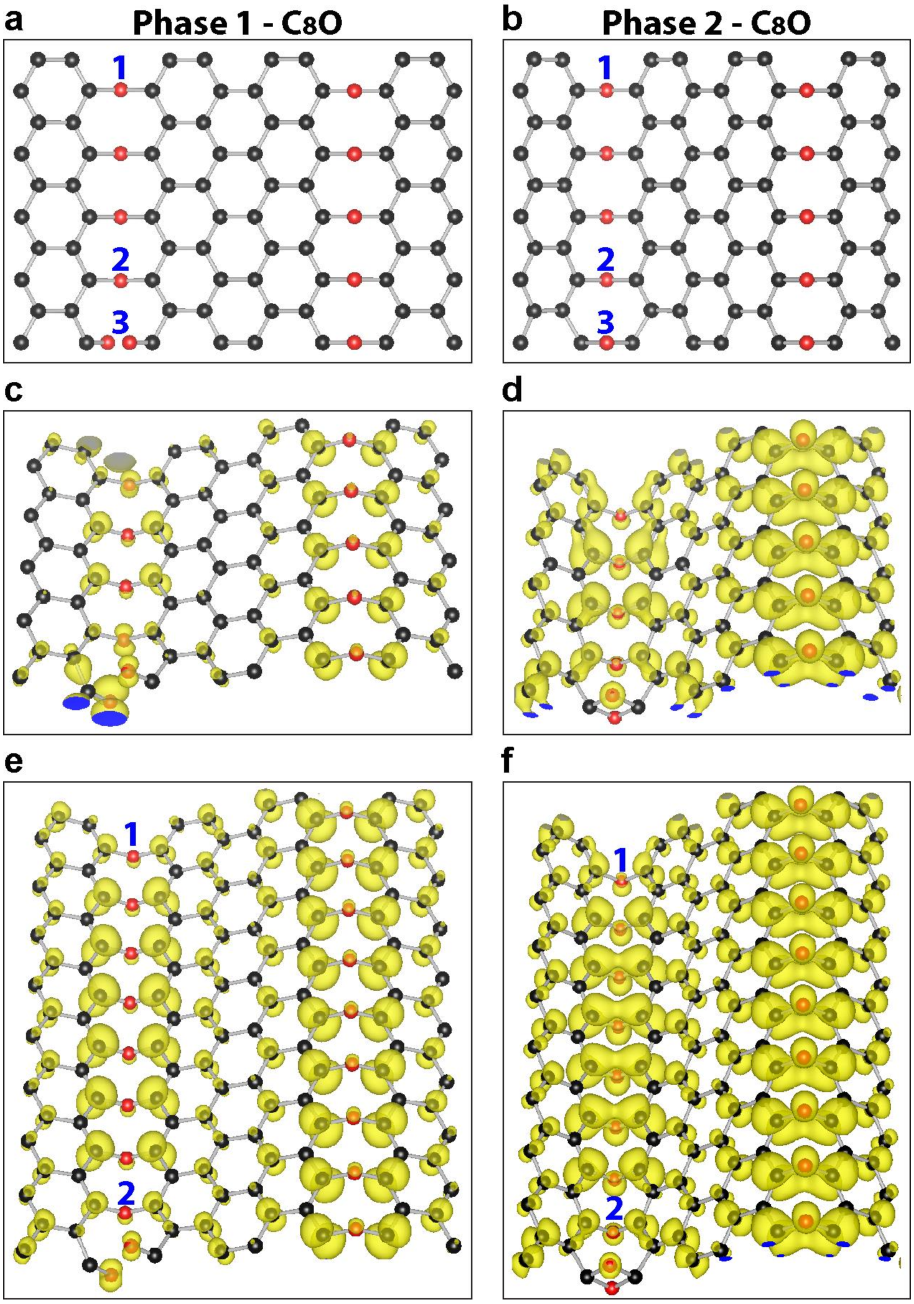

Supplementary Figure 15: Phase 1 and phase 2 C_8O GO with defects by introducing excess oxygen atoms on the epoxy line. The excess doping position is at site 3 for Phase 1 supercells (a) and Phase 2 supercells (b). The perfect epoxy line length is measured by the length between site 1 and site 2. Note that the lattice constant *a* for perfect P1&P2-C_8O GO is 18.384 and 15.718 Å. (c) & (e) Partial charge density results of P1-C_8O 1x5 and 1x9 supercells (within the energy range from -1.0 to 0.0 eV with reference to E_F), respectively. Clearly a carboxyl pair forms at site 3. The fully relaxed lattice constant *a* is 18.341 and 18.352 Å, respectively. (d) and (f) Partial charge density results of P2-C_8O supercells (within the energy range from -1.0 to 0.0 eV with reference to E_F), respectively. At site 3, an epoxy pair is formed instead. The fully relaxed lattice constant *a* is 15.654 and 15.679 Å,

respectively. The signature *lp*-π orbital charge density can be seen beyond the first nearest neighbour of defect. The crystal structure and partial charge density results indicate that this defect only affect the structure locally and it almost has no effects as long as concentration lower than 20% (the length of perfect epoxy line equals or larger than 9.9 Å). The length of perfect epoxy line is measured to be 9.9 and 17.723 Å for these two different size supercells.

Supplementary Table 1: Analysis of chemical bonding in the bi-stable phases C_8O. The orbital hybridization of oxygen and its neighbouring carbon atoms; Partial charge density correspond to the four energy level regions in the DOS of Phase 1 and Phase 2 (as shown in Supplementary Figure 2 (c, d)). The isosurface value is in unit of electron per bohr.

	Orbital Hybridization	Phase 1	Phase 2
1	**Oxygen:**<br>*sp*^{2} for C-O bond<br><br>**Carbon:**<br>*sp*^{2} for C-O bond<br>*sp*^{2} for C-C bond<br><br>Isosurface level: 0.02	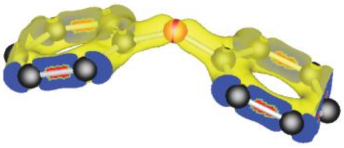	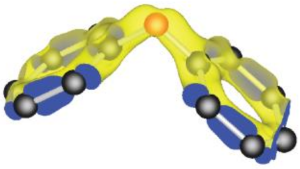
2	**Oxygen:**<br>*sp*^{2} lone pair<br><br>**Carbon:**<br>*sp*^{2} for C-C bond<br><br>Isosurface level: 0.02	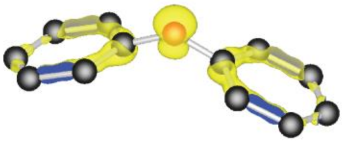	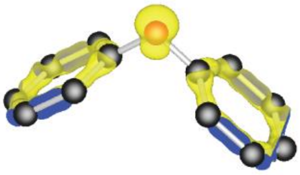
3	**Oxygen:**<br>p_y lone pair<br><br>**Carbon:**<br>π obitals<br><br>Isosurface level: 0.01	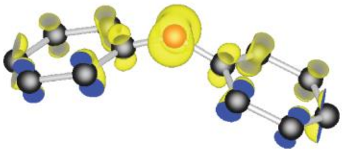	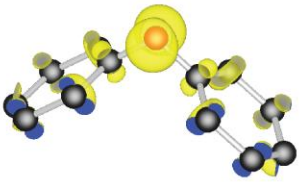
4	**Oxygen:**<br>*sp*^{2} lone pair<br><br>**Carbon:**<br>π obitals<br><br>Isosurface level: 0.003	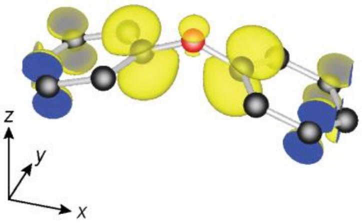 	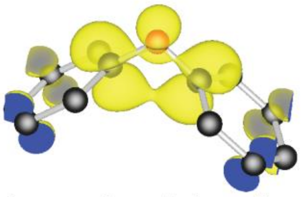<br>***lone pair - π interactions***

Supplementary Table 2: Equilibrium lattice constant *a* and interface energy values of $(C_8O)_4$ and $(C_8O)_6$ supercells with different types of Phase 1 and Phase 2 mixing. (a) Five different phase mixture cases in $(C_8O)_4$ supercells. (b) Seven different phase mixture cases in $(C_8O)_6$ supercells. Note that C_8O is a two-dimensional material. It is controversial to define its thickness. Thus, the interface energy value is expressed as energy per unit length.

a

4 supercells	Crystal	Interface Energy (meV per Å)	Lattice Constant *a*
**1**	$4 \times P1$		73.53
**2**	$3 \times P1 + 1 \times P2$	12.69	70.90
**3**	$2 \times P1 + 2 \times P2$	12.67	68.22
**4**	$1 \times P1 + 3 \times P2$	12.35	65.54
**5**	$4 \times P2$		62.87

b

6 supercells	Crystal	Interface Energy (meV per Å)	Lattice Constant *a*
**1**	$6 \times P1$		110.30
**2**	$5 \times P1 + 1 \times P2$	10.53	107.62
**3**	$4 \times P1 + 2 \times P2$	8.21	104.88
**4**	$3 \times P1 + 3 \times P2$	11.81	102.30
**5**	$2 \times P2 + 4 \times P2$	11.61	99.52
**6**	$1 \times P1 + 5 \times P2$	8.73	96.96
**7**	$6 \times P2$		94.31

Supplementary Note 1: Analysis of chemical bonding in the bi-stable phases C_8O.

Energy level region 1:

- Two half-filled sp^2 orbitals of oxygen atom interact with sp^2 orbitals of its neighbouring carbon atoms to form C-O σ bond.
- sp^2 orbitals of carbon atoms interact with each other to form C-C bond.

Energy level region 2:

- Isolated sp^2 lone pair of oxygen within the C-O-C plane.
- Weak sp^2 orbitals of carbon atoms interact with each other to form C-C bond.

Energy level region 3:

- Isolated p_y lone pair of oxygen perpendicular to the C-O-C plane.
- π bond of carbon atoms.

Energy level region 4:

- Phase 1 - Isolated sp^2 lone pair of oxygen and π orbitals of carbon atoms.
- Phase 2 - sp^2 lone pair of oxygen interacts with π orbitals of its neighbouring carbon atoms.

Supplementary Note 2: Evidences of Phase 3 for C_8O and C_4O at E-field of **0.3 eV per Å.**

For C_8O (Supplementary Figure 5a and 5b), the DOS of P3 suggests that there is a clear band gap near the E_F, which is different from the DOS results of P1 and P2 as shown in Supplementary Figure 2a and 2b. The PDOS and partial charge density of P3 also indicates an entirely different electronic structure in comparison to P1 and P2 shown in Fig. 1d and 1e. It is worth to point out that P3 only exists under **E**-fields. When a sufficiently large **E**-field is applied to the crystal, it seems that the grey shaded π electrons peak of oxygen-bonded carbon atom in P1 shifted to the right and became empty π^* electrons above E_F. The π^* electrons of oxygen-boned carbon atoms is then found to overlap with sp^2 *lp** of oxygen atom in the LUMO region as long as $E > 0.2$ eV per Å. Different from both P1 and P2, Supplementary Figure 5e reveals that oxygen atom of P3 has strong p_y orbital in the HOMO region.

Similarly, the DOS, PDOS and partial charge density results (Supplementary Figure 5c, 5d and 5f) also suggest the existence of P3 for C_4O upon the applied **E**-field.

Supplementary Note 3: Total energy *vs*. displacement of the mid-point of a $(C_8O)_4$ supercell with P1/P2 ratio of 2:2.

This section is devoted to prove feasibility of the two-way actuator design in the main text. In Fig. 6, we propose that the **E**-field induced contraction of P1 could generate adequate mechanical force to stretch P2 and trigger phase transition from P2 to P1 simultaneously. In DFT simulations, it is impossible to apply local **E**-field to one portion of a supercell. Therefore, a theoretical model is presented here.

As shown in Supplementary Figure 9, the supercell $(C_8O)_4$ includes P1 and P2 segments. Total energy of the whole system should be a summation of the total energy of both segments and the interface energy. By assuming an **E**-field strength of 0.5 eV per Å is locally applied to the P1 segment,

the energy of P1 segment as a function of its length shrinkage *d* (i.e., displacement of the interface) is shown in Supplementary Figure 9b, which is obtained by a separated DFT calculation of a $(C_8O)_2$ supercell including only P1 under an **E**-field strength of 0.5 eV per Å. The total energy of the P2 segment as a function of *d* can be obtained from Fig. 1b. Note that the result in Fig. 1b is for C_8O unit cell, whereas the P2 segment of supercell in Supplementary Figure 9 includes two C_8O units. Thus, the total energy in Fig. 1b should be doubled to obtain the results in Supplementary Figure 9b. Adding these two curves in Supplementary Figure 9b together, we should obtain total energy of the whole system as a function of *d*. Here the interface energy is assumed a constant and it will not change the trend of total energy *vs*. *d* relation. The energy monotonously decreases with the increase of *d*, indicating that the motion of mid-point should spontaneously happen. There is no energy barrier to prevent the phase transition from P2 to P1. In others words, by applying an **E**-field locally to the P1 segment, the resultant phase transition from P1 to P2 can supply sufficient mechanical force to trigger simultaneous phase transition from P2 to P1 of the P2 segment.

Supplementary Note 4: Substrate effects on shape memory properties.

Supplementary Figure 11 shows the supercells including P1 and P2 phases of C_8O and a graphene ribbon substrate. After fully relaxation in DFT calculation, both structure are stable. The lattice constant values are 17.8 and 15.6 Å, which are very close to the P1 and P2 of C_8O. The partial charge also reveals negligible difference. The total energy of these two cases are -250.359 and -250.350 eV, respectively. A single point calculation under an external **E**-field of 0.3 eV per Å yield their total energy values of -250.428 eV and -250.626 eV, respectively. The energetic order is swapped. This calculation confirms that phase transition between P1 and P2 of C_8O crystals with substrate can be triggered by the external electric field and thus leads to the shape memory effect.

Supplementary Note 5: Influence of defects on the epoxy lines.

To examine the influences of defects such as oxygen vacancy and redundant oxygen adatom on the structure stability, supercells of P1 and P2 C_8O with size of 1x5, 1x7, and 1x9 were created. They were fully relaxed in our DFT calculations. Supplementary Figure 14 and 15 show the relaxed crystal structure for the 1x5 and 1x9 cases. Note that the 1x7 supercells have very similar results to those of 1x9 cases. Supplementary Figure 14 and 15 (a and b) shows the top view of the 1x5 supercells, in which the position and configuration of the two types of defects are depicted. Supplementary Figure 14 shows that by removing one oxygen atom in the P2 supercell a C-C bond is formed (bond length approximately 1.43 Å), whereas the C-C bond is elongated to 1.45 Å in the P1 supercell. For the oxygen redundant case, the P2 supercell has an epoxy pair, while a carboxyl pair is formed in P1 supercell. Supplementary Figure 14 and 15 (c-f) summarises the partial charge density of P1 and P2 supercells. Clearly beyond the first nearest neighbour of the defect, the partial charge profiles of the C-O-C angles share the similar features to the perfect P1 and P2 phases. The lattice constants of the supercells, which are listed in the caption of Supplementary Figure 14 and Figure 15, are becoming closer to those of perfect P1 and P2 unit cells along *x*-axis with the reduction of defect concentration, the difference within 1% at defect concentration of 11%. Note that smaller supercells of 1x3 were also tested and we found the P1 phase is not stable in the oxygen vacancy case. Therefore, we can conclude that these two typical defects have limited effects on the structural stability of P1 and P2 at a defect density smaller than 20%.

Supplementary References

1 Dove, Martin T. Introduction to lattice dynamics, Cambridge university press. (1993).

2 Leng, J., Lan, X., Liu, Y. & Du, S. Shape-memory polymers and their composites: stimulus methods and applications. *Progress in Materials Science* **56**, 1077-1135 (2011).

3 Henry, C., McKnight, G., Enke, A., Bortolin, R. & Joshi, S. 3D FEA simulation of segmented reinforcement variable stiffness composites. *Proceedings of SPIE* **6929**, 692920X1- 69290X12 (2008).
